# Cognitive Impairment after Post-Acute COVID-19 Infection: A Systematic Review of the Literature

**DOI:** 10.3390/jpm12122070

**Published:** 2022-12-15

**Authors:** Andrea Perrottelli, Noemi Sansone, Giulia Maria Giordano, Edoardo Caporusso, Luigi Giuliani, Antonio Melillo, Pasquale Pezzella, Paola Bucci, Armida Mucci, Silvana Galderisi

**Affiliations:** Department of Psychiatry, University of Campania “Luigi Vanvitelli”, Largo Madonna delle Grazie, 80138 Naples, Italy

**Keywords:** COVID-19, SARS-CoV-2 coronavirus, long covid, cognition, cognitive dysfunction, executive functions, attention, memory

## Abstract

The present study aims to provide a critical overview of the literature on the relationships between post-acute COVID-19 infection and cognitive impairment, highlighting the limitations and confounding factors. A systematic search of articles published from 1 January 2020 to 1 July 2022 was performed in PubMed/Medline. We followed the Preferred Reporting Items for Systematic Reviews and Meta-Analyses (PRISMA) guidelines. Only studies using validated instruments for the assessment of cognitive impairment were included. Out of 5515 screened records, 72 studies met the inclusion criteria. The available evidence revealed the presence of impairment in executive functions, speed of processing, attention and memory in subjects recovered from COVID-19. However, several limitations of the literature reviewed should be highlighted: most studies were performed on small samples, not stratified by severity of disease and age, used as a cross-sectional or a short-term longitudinal design and provided a limited assessment of the different cognitive domains. Few studies investigated the neurobiological correlates of cognitive deficits in individuals recovered from COVID-19. Further studies with an adequate methodological design are needed for an in-depth characterization of cognitive impairment in individuals recovered from COVID-19.

## 1. Introduction

Since the beginning of the coronavirus disease 2019 (COVID-19) pandemic, caused by Severe Acute Respiratory Syndrome Coronavirus 2 (SARS-CoV-2), the global case count has reached nearly 616 million as of October 2022 [1,2]. The clinical presentation of COVID-19 is heterogeneous, since the infection can be asymptomatic in some subjects or lead to a severe pulmonary pathology in other patients [3]. Although COVID-19 is mostly known for its dramatic respiratory manifestation, it is often referred as a multisystemic condition, due to its effects on the cardiovascular, osteoarticular, hematopoietic and nervous systems [1,4]. Furthermore, in addition to the symptoms occurring during the acute stage, many patients also complain of long-term sequelae, even after recovery from the infection [5]. This clinical picture is called “long-term COVID-19 syndrome”, and it refers to those symptoms that can be recorded more than 12 weeks after the initial ones [6,7,8].

Neuropsychiatric symptoms such as depression, insomnia, anxiety and cognitive impairments are reported as some of the most persistent, debilitating and concerning alterations in the lives of patients that can develop even after the resolution of the infection [9,10,11,12,13,14,15,16,17,18]. The emergence of these symptoms, observed in numerous COVID-19 patients, is also in line with the outcomes of other respiratory viral infections. For instance, previous coronavirus outbreaks, such as the 2002–2004 severe acute respiratory syndrome (SARS) and the 2012 Middle East respiratory syndrome (MERS), have been commonly associated with cases of confusion during the acute phase of illness and with the onset of neurocognitive impairments or psychiatric symptoms (e.g., depression or anxiety) in the long term [19,20,21,22].

Amongst the different neuropsychiatric symptoms associated with COVID-19, deficits in cognitive functions play a prominent role in hindering a full recovery of patients. Physiologically, these functions comprise different abilities, such as working memory, attention/vigilance, verbal/visual learning, reasoning/problem solving and executive functioning [23,24,25,26]. The presence of cognitive deficits can often lead to substantial detriments to the quality of life and daily functioning of individuals, as also observed in elderly people and other neurological or psychiatric conditions [23,27,28,29,30,31,32,33]. As a matter of fact, people with cognitive impairment might present difficulties in instrumental activities of daily living, in making decisions that affect their everyday activities (medical decisions and managing their finances) and in learning new things and completing tasks that rely heavily on memory and complex reasoning [34,35,36,37]. Unfortunately, no effective treatment is currently available to ameliorate cognitive deficits, since the pathophysiological mechanisms subtending them are not very clear. As a matter of fact, multiple hypotheses have been formulated to explain the pathophysiological processes at the core of these symptoms. Studies seem to highlight that both the direct damages of the virus on the central nervous system and the state of hyperinflammation associated with the infection could trigger their onset [9,10,38]. Additionally, when the coronavirus particles pass the blood–brain barrier and reaches the central nervous system (CNS), glial cells such as astrocytes and microglia are the main targeted cells. This might lead to further exacerbation of the neuronal immune system and to subsequent neuronal damages [38].

Furthermore, the entity of the cognitive impairment might be strongly correlated to the duration and the severity of the respiratory pathology, probably due to the persistent and prolonged state of inflammation that certain patients experience [23,39,40]. However, despite the large number of studies currently available, we are still unable to precisely determine the pathophysiological bases of these aspects or the pathological features of the disease that trigger their development.

Given the high incidence of COVID-19 globally and the concern associated with long-term consequences of the infection, data on the cognitive impairment linked to SARS-CoV-2 infection has been published at a high rate. We hypothesize that cognitive impairments will be reported in the majority of the studies included in the review. However, a precise clinical characterization of these deficits might not be as clear yet due to methodological limitations. Therefore, a careful screening of the current literature would provide some suggestions on how future studies should be designed and implemented to obtain a precise characterization of these symptoms. 

The aim of the current systematic review is to provide an extensive overview on the published studies on the topic, with a focus on: (1) the characteristics of the samples; (2) the different types of cognitive assessment scales used; (3) the main findings concerning the cognitive impairments reported in COVID-19 patients; (4) the potential limitations of the studies and some possible approaches to overcome them.

## 2. Materials and Methods

A systematic literature search of the articles published between 1 January 2020 and 1 July 2022 using the PubMed database was undertaken following the Preferred Reporting Items for Systematic Reviews and Meta-Analyses (PRISMA) guidelines [41].

The following combination of search terms was used:

“(COVID-19 OR SARS-COV-2 OR 2019-nCoV OR “long covid” OR “persistent covid” OR “post covid” OR “long-haul covid”) AND (cognition OR neurocognition OR “cognitive deficit” OR “cognitive impairment”)”.

The inclusion and exclusion criteria set for the search aimed at considering research papers that characterized cognitive impairments through a standardized and objective methodology.

Therefore, the inclusion criteria for the studies retrieved were the following: (1) studies that assessed at least one cognitive domain through standardized tests or test batteries, or studies that evaluated cognitive functions through short screening tools, such as the Mini Mental State Examination (MMSE) or the Montreal Cognitive Assessment (MoCA), which are widely implemented in everyday clinical practice and provide a total score of cognitive performance (global rating for cognition); (2) studies that included subjects over 18 years old.

The exclusion criteria were the following: (1) studies that were not in English; (2) no full-text available; (3) studies evaluating cognition through subjective scales or questionnaires, which can often lack in reliability; (4) studies reporting cognitive deficits as “brain fog” or “confusion”, which do not allow an objective evaluation and quantification of the presence of cognitive impairments.

Two researchers (AP and NS) independently screened for eligibility all the articles by titles and abstracts and then proceeded to read the full text. Discrepancies in the selection of the eligible articles were discussed in advance with the whole group and were resolved by discussion and consensus.

## 3. Results

### 3.1. Search Results and Characteristics of Studies

**Figure 1:**
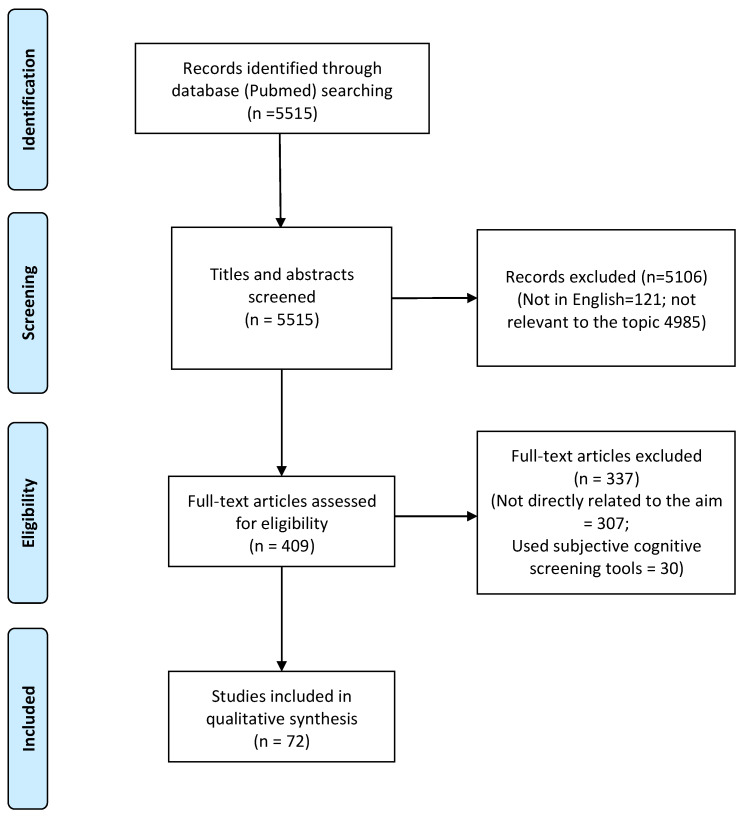
PRISMA flowchart of the included studies.

The literature search yielded 5515 articles (Figure 1). The studies were screened by titles and abstracts: 121 were excluded because they were not in the English language, while 4985 were not relevant to the topic. Therefore, 409 full-text articles were assessed for eligibility. Of these articles, 307 were excluded because they were not directly related to the aim of the review, while 30 were excluded because they used subjective assessment tools to assess cognition. Thus, a final number of 72 studies were included in the current systematic review. The characteristics of the studies included, such as sample size, cognitive domains assessed, assessment instruments, main results and limitations of the study, are all listed in Table S1 (Supplementary Materials).

The PRISMA diagram details the search and selection process applied during our systematic literature search and review.

The studies included five systematic reviews, two of which also performed a meta-analysis, and three narrative reviews. The reviews were considered as part of the included articles (Table S1) and to confirm that no article was missed from the PubMed search.

Thirty cohort studies and twenty-two cross-sectional studies were also included. Finally, among the other study designs, we included two case-control studies, two case reports and six case series studies.

### 3.2. Sample Characteristics

#### 3.2.1. Size, Inclusion Criteria and General Design Characteristics of the Studies

The number of subjects included varied significantly among the studies: the highest sample size was 1539 patients [42], while the lowest was 12 [43], not considering case reports and case series [43,44,45,46,47,48,49,50,51,52]. Most of the studies included a number between 60 and 100 patients [53,54,55,56,57,58], but several articles reported a smaller sample size [9,40,59,60,61,62,63,64,65].

Amongst the most frequent inclusion criteria to recruit patients, the studies always reported at least a positive diagnosis for SARS-CoV-2, confirmed by the polymerase chain reaction (PCR) test, and in some cases, the onset of pulmonary complications, such as dyspnea, desaturation or the presence of chest radiographs reporting lung damage. Furthermore, four studies specifically considered the presence of psychiatric, neurological and severe internal comorbidities in the included subjects that could have affected the cognitive evaluation [66,67,68,69]. Moreover, only 17 studies included a control group, represented by subjects who were negative to a COVID-19 diagnosis [9,42,43,55,57,59,62,63,65,66,67,69,70,71,72,73,74]. Most of the control subjects, who were compared to the COVID-19 patients, were matched by age, gender and education levels but not for preexisting comorbidities. Furthermore, amongst all the included studies, only a few of them evaluated specifically patients shortly after acute infection [61,75,76,77], while the rest performed evaluations after 12 weeks or more. Finally, most of the studies included were performed in Germany, Italy, the USA and China. 

#### 3.2.2. Age and Gender

The ages of the included subjects varied largely: most of the studies (N = 44) reported a mean age between 50 and 60 years [40,48,49,53,58,62,64,74,78,79,80,81,82,83,84,85,86,87,88,89,90,91,92,93,94] or between 60 and 70 years [42,43,44,45,47,56,59,60,63,67,68,75,76,77,95,96,97,98,99]; only five studies considered people with a mean age between 20 and 40 years [46,54,57,70,72], while eight studies included subjects with a mean age between 40 and 50 years [9,50,51,55,61,65,73,100]. It is worth noting that six of the studies performed evaluations on samples of a mean age of 70 years and over [52,66,69,101,102,103]. It appears that the oldest subject recruited was 96 years old [102]. Furthermore, the majority of studies enrolled a sample primarily composed by male subjects, with a M/F ratio spanning between 52% and 83.3% [40,43,44,45,47,48,52,58,59,60,62,63,65,67,68,73,74,75,76,77,78,79,80,82,83,86,88,89,90,91,92,93,94,95,96,97,99,101,102,103] (Table S1).

#### 3.2.3. Hospitalization Rates and Severity of the Respiratory Pathology

In regard to the severity of the disease, 36 of the studies included samples composed by patients that were all hospitalized due to complications of the infection [40,43,44,45,46,47,48,49,51,52,53,58,59,60,62,67,68,72,74,75,76,78,79,82,83,86,87,89,90,91,93,95,96,97,99,102]. Seven of the studies included more than 50% of hospitalized patients [9,63,80,84,88,92,98]. Amongst these populations, 11 studies included patients who experienced severe respiratory symptoms requiring either admission in the intensive care unit (ICU) and intubation or oxygen therapy [53,60,75,77,78,82,90,93,95,97,99]. 

On the other hand, six of the studies performed the evaluations on asymptomatic patients or on those who did not need admission into a COVID-19 ward due to mild respiratory complications [54,55,57,70,81,103]. 

Finally, only a few studies (N = 4) stratified the sample into different degrees of severity, depending on the use of oxygen therapy or mechanical ventilation [9,71,82,94].

### 3.3. Evaluation of Cognitive Impairments in COVID-19 Patients: Assessment Instruments and Main Results

#### 3.3.1. Assessment Instruments

The included studies employed several different assessment instruments to evaluate the presence of cognitive impairments in COVID-19 patients. The majority of the studies used screening tools that provide only a global score of cognitive functioning. On the other hand, some studies used specific tests that allow the evaluation and characterization of subjects’ performances in single cognitive domains. As for the tools used to obtain a global score of cognitive functions, several authors chose clinical screening tools. In fact, 29 studies used the Montreal Cognitive Assessment (MoCA) [43,47,48,49,50,56,57,59,67,69,70,74,75,77,79,80,81,82,83,84,85,92,95,96,97,98,99,101,102], and 14 used the Mini Mental State Examination (MMSE) [44,49,50,51,60,62,63,66,68,73,79,90,101,103]. Twelve out of the twenty-nine studies using the MoCA [43,47,50,59,69,79,81,83,84,95,96,101] and ten out of the fourteen studies using MMSE [44,50,51,60,62,63,66,73,79,101] complemented the screening tools with other assessment instruments specific for single cognitive domains. 

As for the evaluation of single cognitive domains, the tests employed were more numerous and heterogeneous (Table 1). The tests consistently used throughout the studies were the digit span forward and reverse test for the assessment of working memory and attention and the Trail-Making Test for executive functions and speed of processing.

#### 3.3.2. Main Results of Cognitive Assessment

A global cognitive impairment was found in 31 studies of the assessed COVID-19 patients [40,49,50,53,55,57,58,60,61,62,63,65,66,67,69,71,75,76,78,79,81,83,84,86,88,91,96,98,99,101,104]. Only eight studies reported no significant global cognitive impairments in the included samples of COVID-19 patients [51,70,73,85,90,93,97,98]. 

Studies that reported results on single cognitive domains allowed us to draw a more detailed overview of the deficits present in this clinical population. The most affected cognitive domains were the executive functions, memory and speed of processing. In fact, of the 50 studies assessing executive functions through specific tests, 26 reported a significant deficit in executive functions in COVID-19 patients [40,43,45,46,47,53,54,57,59,60,61,62,63,65,69,71,74,75,81,84,85,88,91,96,100,101]. These deficits might include difficulties in planning, abstraction, behavioral control and orientation.

The memory domain was assessed individually in 37 studies and resulted to be compromised in 30 of them. Within this domain, the most impaired aspects were working memory (N = 17) [40,43,45,53,55,58,59,61,69,76,78,83,84,89,91,96,100], visuospatial memory (N = 6) [50,63,69,73,81,84] and episodic memory (N = 4) [54,60,69,91]. All the aspects of memory that were considered in the studies included, from storage and the maintenance of new information to the recollection of old ones, seem to be impaired.

As for the attention domain, it was evaluated in 23 studies and resulted to be compromised in 10 of them [46,51,55,61,75,76,78,84,89,100]. Moreover, patients showing impairment in a single cognitive domain often presented deficits in attention [84]. 

Another cognitive domain severely affected following SARS-CoV-2 infection was the speed of processing that were impaired in 9 studies [46,54,63,65,76,81,83,91,100] out of 26. This deficit is characterized by an overall decrease in cognitive speed and ability to concentrate and is often subjectively recognized by patients as a remarkable modification in their abilities following COVID-19 infection, as frequently reported in subjective questionnaires administered alongside the objective assessments [54,55,92,93].

Finally, twelve studies assessed verbal learning, and all of them reported poor performance [52,53,59,61,62,63,69,78,81,83,84,96].

### 3.4. Outcomes of Neuroimaging and Neuroinflammatory Studies

In addition to the cognitive assessment, some of the studies also collected imaging data to explore potential neurobiological correlates of cognitive deficits. In particular, eight studies employed electroencephalographic (EEG) recordings [46,49,55,56,59,63,75,85], ten studies used magnetic resonance imaging (MRI) [46,47,55,56,63,74,75,85,86,96], two studies employed fluorodeoxyglucose positron emission tomography (FDG-PET) [81,96] and three studies collected brain computerized tomography (CT) scans [46,75,85].

One study that included EEG recordings found abnormalities in two out of the twelve individuals that reported cognitive impairment following SARS-CoV-2 infection [56]. The EEG recordings were characterized by the presence of slow and sharp waves across the left temporal region and of the prominent slowing of cerebral activity in the right temporal lobe, as observed by qualitative evaluation of the EEG recordings of these two patients [56]. No other significant findings emerged in the EEG recordings of other studies [46,49,55,56,59,63,75,85].

As for the MRI studies, one study found that subjects with cognitive impairment, particularly with executive deficits, also presented microvascular events following an acute hypercoagulable state and chronic neuroinflammatory processes [74]. Another study found that the mean score in the visuospatial memory test was decreased in patients presenting white matter abnormalities, especially in the frontal and parietal lobes [86]. Conversely, other studies did not find any remarkable alterations in the structural and functional neuroimaging indices in COVID-19 patients [46,47,55,56,63,74,75,85,86,96]. As for the FDG-PET results, one study found a significant correlation between frontoparietal hypometabolism and a lower general cognitive performance, as assessed using the MoCA [96]. As to the CT findings, no alteration was found in COVID-19 patients with cognitive impairment [46,75,85].

As an additional approach, other studies also employed blood exams to trace specific associations between biomarkers and cognitive deficits. Ten studies analyzed c-reactive protein (CRP) [9,40,43,55,65,69,74,82,84,98], and eight studies measured d-dimer levels [9,40,55,61,69,82,84,98]; however, only a few studies specifically correlated the levels of these biomarkers to the presence of cognitive deficits, and the results were sparse and inconsistent. In particular, a relationship between the CRP levels and attention impairment was found [65], as well as between the d-dimer levels and verbal fluency, verbal memory and psychomotor speed impairment [40]. However, a lack of association between CRP, d-dimer and also ferritin and IL-6 with neurocognitive deficits was reported [9].

## 4. Discussion

The current review shows how the cumulative evidence in the literature highlights an association between SARS-CoV-2 infection and the onset of cognitive deficits. Nevertheless, the neurobiological and clinical correlates of these deficits are still unclear. 

The following sections of the discussion will provide a critical commentary on the results presented in the present review of the literature, including an overview of the main findings concerning the appearance of cognitive deficits in COVID-19 patients and the possible methodological aspects of the included studies that might have affected the results, in addition to the possible pathophysiological explanations that might lead to their appearance.

### 4.1. Main Findings and Characteristics of the Study Design and of Included Samples: Possible Bias of the Collected Outcomes

#### 4.1.1. Main Findings and Methodological Limitations

Numerous studies have reported alterations in cognitive functioning in the evaluation of COVID-19 patients both during the acute phase or even for a prolonged period following recovery from infection [40,49,50,53,55,57,58,60,61,62,63,65,66,67,69,71,75,76,78,79,81,83,84,86,88,91,96,98,99,101,104]. The presence of cognitive deficits was mostly evaluated through the MoCA and MMSE scales; several of these studies reported that a vast number of the COVID-19 patients obtained a lower score than the cutoff value, flagging the presence of a generalized cognitive impairment. On the other side, studies that employed tests for specific cognitive domains were less numerous but provided interesting insights in the characterization of the cognitive impairment associated with COVID-19. The most affected cognitive domains were found to be the executive functions, speed of processing, memory and attention.

The onset of these deficits, which are often described by the subjects as highly debilitating, can remarkably affect their quality of life by interfering with everyday activities [12,14,34,105,106,107].

One of the main limitations in the design of the included studies is that the majority of them did not take into account cognitive deficits preceding the COVID-19 infection. Most of the studies attempted to retrieve the missing information about the cognitive status before the infection through subjective assessments obtained through interviews with patients and relatives. This may have produced recall bias, since potential preexisting cognitive deficits may have been overlooked by patients and their relatives. However, one study provided pre-infection MoCA evaluations of a nested sample of 52 patients from the population of Atahualpa, a cluster of community dwellers living in rural Ecuador [56]. In this study, post-COVID cognitive evaluations reported lower scores in 21% of the seropositive cohort compared to pre-COVID evaluations. Conversely, in seronegative controls, only 2% reported a worsening in MoCA scores, confirming the hypothesis of alterations in cognition after the infection [56]. Furthermore, the influence of confounding factors affecting cognition, especially during the acute phase of the infection, has often been overlooked. For instance, symptoms of depression and anxiety, which could be connected to the distress experienced during hospitalization, might have appeared as well, leading to fluctuations in the cognitive performance [108]. Finally, in addition to the lack of pre-COVID-19 assessments, the majority of the protocols lacked follow-up assessments, which did not allow to verify the persistence over time of the deficits.

Another main limitation in the experimental design was the heterogeneity of the inclusion criteria for the patients. The inclusion criteria ranged from the inclusion of patients who only presented a confirmed diagnosis for COVID-19 to subjects who experienced pulmonary complications, requiring hospital admission and respiratory assistance. Furthermore, many of the studies completely lacked a control group. When present, control subjects were defined as subjects who were negative at the time of the evaluations or who never received a diagnosis of SARS-CoV-2 infection; however, these control populations were often not matched to patients for preexisting comorbidities. Another noticeable feature is that there is a lack of studies considering as the control sample subjects who tested positive to COVID-19 but did not present cognitive impairment. Exploring this scenario might advance the knowledge on both common and different features of COVID-19-positive patients with and without cognitive impairment, such as the severity of the respiratory symptoms experienced, demographic data, environmental factor or comorbidities with psychiatric disorders [109,110]. It is certainly important to consider that the majority of studies was developed in the core period of the pandemic, which might have led to potential difficulties in designing methods and used less strict inclusion criteria in order to favor quick data collection [111].

Some considerations need to be formulated regarding the instruments used for the evaluations of cognitive impairment. The most frequent instruments used were screening tools, such as the MoCA and MMSE. Due to the high number of subjects reporting these deficits following COVID-19 infection, the use of MoCA and MMSE, which, in 10–15 min, provide a general assessment of cognitive functioning, should be strongly encouraged in the clinical practice of COVID-19 patients as a routine screening test. However, certain limits of these scales should be considered for the full characterization of cognitive impairments. Previous uses of these tools in clinical and research settings have showed that MoCA may be a better tool to detect mild cognitive deficits [112], while MMSE is helpful in identifying severe impairment and dementia [113]. Therefore, studies using only the MMSE might have missed the detection of mild alterations in cognitive functioning. Furthermore, both tools do not provide a detailed characterization of the impairment, since they produce only a general cognitive functioning score. Therefore, information obtained by screening tools should be integrated with that evaluated with cognitive batteries in order to guarantee a complete analysis of the cognitive domains and subdomains. In addition, the extreme variability among the assessing tools does not allow to clearly summarize the results for a single cognitive domain. Furthermore, several studies performed evaluations in telemedicine, due to containment measures and lockdown imposed by the outbreak of the pandemic, which might have affected the quality of data collection due to the difficulty of sustaining attention online, the weakness of online bonds and the weak commitment to online assessments [114]. Finally, only a few studies have incorporated the use of neuroimaging data, so functional and structural changes associated with cognitive impairment are yet to be explored. More studies using a direct correlation analysis of brain imaging findings with cognitive impairments might provide data that could better predict the prognosis and help the prevention of these deficits in clinical settings.

#### 4.1.2. Characteristics of the Subjects Included and Possible Selection Bias

Other elements to be considered are the recruitment bias and consequent imbalance of the included subjects as to gender or age, as well as the sample size, which could have influenced the outcomes.

Firstly, the samples were mostly composed of male adults, and, according to results, men seemed to be the most affected by the changes in cognition. This might be solely due to a selection bias, since more men might have been recruited for the studies since they are believed to be more susceptible to severe COVID-19 pathology. Indeed, males present higher expression of the angiotensin-converting enzyme 2 (ACE2) receptor and the transmembrane protease serine 2 (TMPRSS2), which are targeted by the virus to access the body, leading to a higher chance of experiencing life-threatening respiratory pathology [115]. On the other side, recent studies highlighted that women have a higher prevalence of neuropsychological long-COVID symptoms than men [116]. This might be linked to specific hormonal factors that lead to the elicitation of stronger and prolonged immune response due to the higher production of IgG antibodies [117,118] or to greater self-awareness in women compared to men on changes in health [116]. However, it is still not possible to confirm whether the onset and severity of cognitive dysfunctions due to COVID-19 are influenced by gender. Future studies should include a greater balance of male and female patients or at least use a statistical analysis that controls for sex as a confounding variable. Furthermore, it might be interesting to examine if the levels of inflammatory markers are affected by gender and correlate to the severity of the cognitive impairments. 

A second factor that must be considered while reviewing the studies is the age of the included subjects. Most of the studies performed assessments on samples with a mean age comprised between 50 and 70 years. One meta-analysis found that MoCA score progressively decreased for each increase of 1 year in age, suggesting that aging usually correlates with a progressive deterioration in cognitive functions [24]. Therefore, the potential cognitive impairment found in elderly people with COVID-19 could be due to aging effects [119,120]. However, studies assessing cognition in younger patients (mean age between 20 and 40 years) found the presence of cognitive impairments such as in studies that included older subjects [57,70,86] Furthermore, one meta-analysis found no significant differences in cognitive impairment between age groups, suggesting that these deficits cannot be solely attributed to the consequences of aging [23].

Another main limitation of the reviewed literature is that the study samples were often small, heterogeneous and not stratified by education level and, most importantly, by disease severity, a limitation reported by 48% of the studies.

In light of the above limitations, further investigations on the topic should include a longitudinal examination of subjects, comparisons between COVID-19 patients and control groups and should try to control for confounding factors. In particular, a stratification of participants according to variables such as gender, age, level of education and disease severity would provide more generalizable results on the relationship between COVID-19 infection and cognitive deficits. 

### 4.2. Cognitive Impairment in COVID-19: Possible Pathophysiological Mechanisms

One of the main objectives of the studies considered was also to unveil the biological underpinnings and to trace the associations between the clinical manifestations of COVID-19 illness and the onset of cognitive deficits, in order to guide more effective prevention and treatment strategies.

The first hypothesis developed by the reviewed studies claimed that the appearance of cognitive impairments was mainly due to the cellular damages caused by the presence of the virus in the nervous system. [115]. However, further insights from clinical and post-mortem studies seem to pinpoint that the abnormal and prolonged hyperinflammatory state following infection might be a more important feature to determine the onset of these symptoms post-infection [74,121]. Previous evidence collected from studies on respiratory non-SARS-CoV-2 viral infections showed that systemic and excessive cytokine inflammatory responses, characterized by prolonged IL-6 and TNF-α expression, can lead to damages in brain areas such as the hippocampus [19,122,123]. This trend was also detected in COVID-19 patients with cognitive impairment in which a prominent inflammatory response is flagged by a generalized increase in the concentration of the biomarkers of inflammation such as CPR and various cytokines [65,98,124,125]. Furthermore, when the coronavirus particles pass the blood–brain barrier and reaches the central nervous system (CNS), glial cells such as astrocytes and microglia are the main targeted cells. Disruption in the activity of the glia has been associated with alterations in the neuroprotective functionality of the neuronal immune system and to subsequent neuronal damages.

Another neurobiological path that has been investigated is the decrease in the enzymatic activity of angiotensin-converting enzyme 2 (ACE 2) observed in COVID-19 infection. ACE 2 is unevenly expressed in the brain areas, and its decrease in specific regions is related to signs and symptoms of neurological damage: low levels of ACE 2 in the cerebral cortex may determine the general neurocognitive alterations, while reduced levels of ACE 2 in the hippocampus could be linked specifically to memory alterations [124,126].

An additional approach in understanding the etiology of these symptoms was to examine how the severity of the other clinical aspects of the COVID-19 pathology could be correlated to the onset of cognitive impairments. For instance, ventilated patients or, more broadly, subjects experiencing severe respiratory pathology or a decrease in their blood oxygen level, were the most affected by cognitive impairment, mainly in memory, executive functions and verbal fluency, in some studies [40,124]. This is in line with the hypothesis that the cognitive deficits could be the result of damages to brain areas particularly sensitive to low oxygenation, such as the hippocampus [61,127,128], which has a key role in memory. However, other studies reported that patients who were admitted to the intensive care unit (ICU) or that received oxygen therapy had lesser impairment in cognitive functions than patients who did not receive these supplementary treatments. This finding might suggest that patients with a less compromised respiratory status were more impaired in cognition compared to patients with more severe breathing difficulties. One hypothesis to explain this outcome was that the use of oxygen supplementation, received during hospitalization, might have prevented the risk of developing brain hypoxia or mild fluctuations in the blood oxygen, which might have been underestimated in patients’ less critical respiratory pathology [68,129]. Furthermore, some studies detected cognitive deficits in subjects displaying very mild respiratory symptoms or even in asymptomatic cases, suggesting that the development of severe breathing pathology may not be a mandatory element to trigger changes in cognition [70].

In summary, it has been hypothesized that the direct damages to neuronal cells (especially glial cells rather than neurons), a state of hyperinflammation induced by SARS-CoV-2 and brain hypoxia due to a low level of blood oxygen, can all lead to cerebral alterations observed in some subjects, which do not seem to be restored even after infection clearance [56,86,96]. These factors might be the primary causes of the onset of cognitive impairments [38,61,124,126,127]. However, more studies should be conducted on the development of cognitive impairment post-COVID-19, including the use of neuroimaging and neuroinflammatory biomarkers and the stratification of the samples according to the severity of the respiratory symptoms in order to better comprehend the pathophysiological determinants of cognitive impairments.

## 5. Conclusions

The current review reinforces the general idea that there is a link between COVID-19 illness and the onset of cognitive impairment. Therefore, since a large subset of subjects affected by COVID-19 presents these cognitive deficits, the implementation of their assessment should become part of the routine clinical practice for these patients. In fact, due to the severe impact of these symptoms on the quality of life, early detection and intervention could improve their resolution in the long run.

However, despite this evident relationship, the current review highlights how there is a need for further studies with: (1) a longitudinal design; (2) an extensive and standardized assessment of the different cognitive domains; (3) representative and balanced samples of subjects and control groups of participants; (4) data on neuroimaging and inflammatory biomarkers.

A greater attention to these methodological issues might help to identify the neurobiological correlates of COVID-19-related cognitive impairment and evaluate their courses over time, leading to the development of preventive and therapeutic strategies for this concerning consequence of the infection.

## Figures and Tables

**Table 1:** List of the tests employed to assess specific cognitive domains.

Cognitive Domain (and Subdomains if Present)		Assessment Scales	Studies
**Executive Functions**		FAB	[43,44,45,46,47,59,60,63,66,101]
		TMT-B	[40,46,50,52,53,54,58,59,61,63,64,65,69,81,84,96,100,101]
		Stroop Color-Word Test	[43,54,59,60,61,84,96,101]
		Tower of London,	[73]
		CDT	[62]
		WCST	[71]
		Similarities test from the Wechsler Adult Intelligence Scale (WAIS IV),	[60]
		Brixton test	[60]
		D-KEFS	[50]
**Speed Of Processing**		TMT-A	[46,50,52,53,54,59,61,63,65,69,81,84,96,100,101]
		SDMT	[49,59,61,63,81,83,96,101]
		SCT	[65]
		SYMBOL SEARCH	[84]
		PASAT	[83]
**Memory**	*Episodic Memory*	FCRST	[69]
		List Learning Test	[54]
		Dubois five words test	[60]
		Logical Memory I e II from the Weschler Memory Scale (WMS-IV)	[91]
		Category Cued Verbal Fluency	[53]
	*Verbal Memory*	Word List Recognition Memory Test	[71]
		VVM	[91]
		Pictorial Associative Memory Test	[71]
	*Visuospatial Memory*	ROCFT/RCFT	[50,69,73,84]
		DRT	[69]
		BVMT-R	[81]
		VOSP	[63]
		NAB (visual discrimination task)	[50]
		SPART	[83]
		2D-Mental Rotation Test	[71]
	*Working Memory*	Digit Span Forward and Reverse	[50,53,59,60,62,63,65,78,81,84,89,96,101].
**Attention/** **Vigilance**		RBANS	[52,86]
		Digit Span Forward and Reverse	[50,53,59,60,62,63,65,78,81,84,89,96,101].
		CPT-II	[65,84]
		Tea Attention Test	[73]
		D2-R Test	[95]
		Attentional Matrices	[66]
		TAP	[91]
		Vigilance Task	[43]
		CVAT	[51]
**Language**	*Global Evaluation*	BNT	[69,84]
		40 Words oral naming test	[60]
		SAND	[63]
		BDAE (Language subtest)	[52]
		Categorical and lexical verbal fluencies during two-minute test	[60]
	*Verbal Fluency*	FAS	[69,78,101]
		Letter Cued Verbal Fluency	[53]
		Semantic Fluency Test	[61,62,81,96]
		Phonemic Fluency Test	[61,96,100]
		Category Fluency Test	[53,59,71,100]
		WLG	[83]
		COWA	[73,89]
	*Verbal Learning*	RAVLT	[53,59,62,63,78,84],
		HVLT-R	[52,81,96]
		TAVEC	[61,69]
		SRT	[83]

BDAE = Boston Diagnostic Aphasia Examination; BMET = Brief Memory and Executive Test; BNT = Boston Naming Test; BVMT-R = Brief Visuospatial Memory Test-Revised; CDT = Clock Drawing Test; COWA = Controlled Oral Word Association by categories; CPM47 = Colored Progressive Matrices 47; CPT = Continuous Performance Test; CVLT = California Verbal Learning Test; CVAT = Continuous Visual Attention Test; D-KEFS = Delis-Kaplan Executive Functions Test; DRT = Digit Retention Test; FAB = Frontal Assessment Battery; FAS = Verbal Fluency Test; FCRST = Free and Cued Selective Reminding Test; FWIT = Color-Word Interference Test; HVLT = Hopkins Verbal Learning Test-Revised; NAB = Neuropsychological Assessment Battery; OMC = Orientation-Memory-Concentration Test; PASAT = Paced Auditory Serial Addition Test; RAVLT = Rey Auditory Verbal Learning Test; RBANS = Repeatable Battery for the Assessment of Neuropsychological Status; RCFT = Rey Complex Figure Test; RDS = Reliable Digit Span; SAND = Screening for aphasia in neurodegeneration; SCT = Sign Coding Test; SDMT = Symbol Digit Modalities Test; SPART = 10/36 Spatial Recall Test; SRT = Selective Reminding Test; TAP = Test of Attentional Performance; TAVEC = Test de Aprendizaje Verbal Espana-Complutense; TMT-A = Trail-Making Test-A; TMT-B = Trail Making Test-B; TMT = Trail Making Test; TSAT = Test of Sustained Attention and Tracking; VVM =Verbal and visual memory test; VOSP = Visual object and space perception battery; WCST = Wisconsin Cart Sorting Test; WLG = Word List Generation Test; WMS-IV = Visual Reproduction of the Wechsler Memory Scale–IV.

## Data Availability

The data presented in the current review are all available within the article and the Supplementary Materials.

## Supplementary Materials

The following supporting information can be downloaded at: https://www.mdpi.com/article/10.3390/jpm12122070/s1: Table S1: Main characteristics of the articles included. References [130,131,132,133] are cited in the supplementary materials.

## References

[1] Miners S., Kehoe P.G., Love S. (2020). Cognitive impact of COVID-19: Looking beyond the short term. Alzheimer’s Res. Ther..

[2] WHO World Health Organization: COVID-19 Dashboard. https://covid19.who.int/.

[3] Leung T.Y.M., Chan A.Y.L., Chan E.W., Chan V.K.Y., Chui C.S.L., Cowling B.J., Gao L., Ge M.Q., Hung I.F.N., Ip M.S.M. (2020). Short- and potential long-term adverse health outcomes of COVID-19: A rapid review. Emerg. Microbes Infect..

[4] Kamal M., Abo Omirah M., Hussein A., Saeed H. (2021). Assessment and characterisation of post-COVID-19 manifestations. Int. J. Clin. Pract..

[5] Islam M.F., Cotler J., Jason L.A. (2020). Post-viral fatigue and COVID-19: Lessons from past epidemics. Fatigue Biomed. Health Behav..

[6] World Health Organization (2021). A Clinical Case Definition of Post COVID-19 Condition by a Delphi Consensus.

[7] Crook H., Raza S., Nowell J., Young M., Edison P. (2021). Long covid—Mechanisms, risk factors, and management. BMJ.

[8] Nalbandian A., Sehgal K., Gupta A., Madhavan M.V., McGroder C., Stevens J.S., Cook J.R., Nordvig A.S., Shalev D., Sehrawat T.S. (2021). Post-acute COVID-19 syndrome. Nat. Med..

[9] Woo M.S., Malsy J., Pöttgen J., Seddiq Zai S., Ufer F., Hadjilaou A., Schmiedel S., Addo M.M., Gerloff C., Heesen C. (2020). Frequent neurocognitive deficits after recovery from mild COVID-19. Brain Commun..

[10] Wu Y., Xu X., Chen Z., Duan J., Hashimoto K., Yang L., Liu C., Yang C. (2020). Nervous system involvement after infection with COVID-19 and other coronaviruses. Brain Behav. Immun..

[11] Ritchie K., Chan D. (2021). The emergence of cognitive COVID. World Psychiatry.

[12] Coleman B., Casiraghi E., Blau H., Chan L., Haendel M.A., Laraway B., Callahan T.J., Deer R.R., Wilkins K.J., Reese J. (2022). Risk of new-onset psychiatric sequelae of COVID-19 in the early and late post-acute phase. World Psychiatry.

[13] De Hert M., Mazereel V., Detraux J., Van Assche K. (2021). Prioritizing COVID-19 vaccination for people with severe mental illness. World Psychiatry.

[14] Li J., Yang Z., Qiu H., Wang Y., Jian L., Ji J., Li K. (2020). Anxiety and depression among general population in China at the peak of the COVID-19 epidemic. World Psychiatry.

[15] Tyrer P. (2020). COVID-19 health anxiety. World Psychiatry.

[16] Wasserman D., Iosue M., Wuestefeld A., Carli V. (2020). Adaptation of evidence-based suicide prevention strategies during and after the COVID-19 pandemic. World Psychiatry.

[17] McIntyre R.S., Lee Y. (2020). Preventing suicide in the context of the COVID-19 pandemic. World Psychiatry.

[18] Rooksby M., Furuhashi T., McLeod H.J. (2020). Hikikomori: A hidden mental health need following the COVID-19 pandemic. World Psychiatry.

[19] Rogers J.P., Chesney E., Oliver D., Pollak T.A., McGuire P., Fusar-Poli P., Zandi M.S., Lewis G., David A.S. (2020). Psychiatric and neuropsychiatric presentations associated with severe coronavirus infections: A systematic review and meta-analysis with comparison to the COVID-19 pandemic. Lancet Psychiatry.

[20] Kępińska A.P., Iyegbe C.O., Vernon A.C., Yolken R., Murray R.M., Pollak T.A. (2020). Schizophrenia and Influenza at the Centenary of the 1918-1919 Spanish Influenza Pandemic: Mechanisms of Psychosis Risk. Front. Psychiatry.

[21] Adhanom Ghebreyesus T. (2020). Addressing mental health needs: An integral part of COVID-19 response. World Psychiatry.

[22] Unützer J., Kimmel R.J., Snowden M. (2020). Psychiatry in the age of COVID-19. World Psychiatry.

[23] Ceban F., Ling S., Lui L.M.W., Lee Y., Gill H., Teopiz K.M., Rodrigues N.B., Subramaniapillai M., Di Vincenzo J.D., Cao B. (2022). Fatigue and cognitive impairment in Post-COVID-19 Syndrome: A systematic review and meta-analysis. Brain Behav. Immun..

[24] Crivelli L., Palmer K., Calandri I., Guekht A., Beghi E., Carroll W., Frontera J., García-Azorín D., Westenberg E., Winkler A.S. (2022). Changes in cognitive functioning after COVID-19: A systematic review and meta-analysis. Alzheimers Dement..

[25] Schou T.M., Joca S., Wegener G., Bay-Richter C. (2021). Psychiatric and neuropsychiatric sequelae of COVID-19—A systematic review. Brain Behav. Immun..

[26] Mucci A., Galderisi S., Green M.F., Nuechterlein K., Rucci P., Gibertoni D., Rossi A., Rocca P., Bertolino A., Bucci P. (2018). Familial aggregation of MATRICS Consensus Cognitive Battery scores in a large sample of outpatients with schizophrenia and their unaffected relatives. Psychol. Med..

[27] Elliott A.F., McGwin G., Owsley C. (2009). Health-related quality of life and visual and cognitive impairment among nursing-home residents. Br. J. Ophthalmol..

[28] Huang L., Li X., Gu X., Zhang H., Ren L., Guo L., Liu M., Wang Y., Cui D., Wang Y. (2022). Health outcomes in people 2 years after surviving hospitalisation with COVID-19: A longitudinal cohort study. Lancet Respir. Med..

[29] Saraçlı Ö., Akca A.S., Atasoy N., Önder Ö., Şenormancı Ö., Kaygisız İ., Atik L. (2015). The Relationship between Quality of Life and Cognitive Functions, Anxiety and Depression among Hospitalized Elderly Patients. Clin. Psychopharmacol. Neurosci..

[30] Tavares-Júnior J.W.L., de Souza A.C.C., Borges J.W.P., Oliveira D.N., Siqueira-Neto J.I., Sobreira-Neto M.A., Braga-Neto P. (2022). COVID-19 associated cognitive impairment: A systematic review. Cortex.

[31] Wlodarczyk J.H., Brodaty H., Hawthorne G. (2004). The relationship between quality of life, Mini-Mental State Examination, and the Instrumental Activities of Daily Living in patients with Alzheimer’s disease. Arch. Gerontol. Geriatr..

[32] Mei Q., Wang F., Bryant A., Wei L., Yuan X., Li J. (2021). Mental health problems among COVID-19 survivors in Wuhan, China. World Psychiatry.

[33] Marazziti D., Stahl S.M. (2020). The relevance of COVID-19 pandemic to psychiatry. World Psychiatry.

[34] Galderisi S., Rossi A., Rocca P., Bertolino A., Mucci A., Bucci P., Rucci P., Gibertoni D., Aguglia E., Amore M. (2014). The influence of illness-related variables, personal resources and context-related factors on real-life functioning of people with schizophrenia. World Psychiatry.

[35] Kuiper J.S., Oude Voshaar R.C., Zuidema S.U., Stolk R.P., Zuidersma M., Smidt N. (2017). The relationship between social functioning and subjective memory complaints in older persons: A population-based longitudinal cohort study. Int J. Geriatr. Psychiatry.

[36] Aretouli E., Brandt J. (2010). Everyday functioning in mild cognitive impairment and its relationship with executive cognition. Int. J. Geriatr. Psychiatry.

[37] Galderisi S., Rucci P., Mucci A., Rossi A., Rocca P., Bertolino A., Aguglia E., Amore M., Bellomo A., Bozzatello P. (2020). The interplay among psychopathology, personal resources, context-related factors and real-life functioning in schizophrenia: Stability in relationships after 4 years and differences in network structure between recovered and non-recovered patients. World Psychiatry.

[38] Li Y.C., Bai W.Z., Hashikawa T. (2020). The neuroinvasive potential of SARS-CoV2 may play a role in the respiratory failure of COVID-19 patients. J. Med. Virol..

[39] Jason L.A., Islam M.F., Conroy K., Cotler J., Torres C., Johnson M., Mabie B. (2021). COVID-19 symptoms over time: Comparing long-haulers to ME/CFS. Fatigue Biomed. Health Behav..

[40] Miskowiak K.W., Johnsen S., Sattler S.M., Nielsen S., Kunalan K., Rungby J., Lapperre T., Porsberg C.M. (2021). Cognitive impairments four months after COVID-19 hospital discharge: Pattern, severity and association with illness variables. Eur. Neuropsychopharmacol..

[41] Moher D., Liberati A., Tetzlaff J., Altman D.G. (2009). Preferred reporting items for systematic reviews and meta-analyses: The PRISMA statement. PLoS Med..

[42] Liu Y.-H., Wang Y.-R., Wang Q.-H., Chen Y., Chen X., Li Y., Cen Y., Xu C., Hu T., Liu X.-D. (2021). Post-infection cognitive impairments in a cohort of elderly patients with COVID-19. Mol. Neurodegener..

[43] Ortelli P., Ferrazzoli D., Sebastianelli L., Engl M., Romanello R., Nardone R., Bonini I., Koch G., Saltuari L., Quartarone A. (2021). Neuropsychological and neurophysiological correlates of fatigue in post-acute patients with neurological manifestations of COVID-19: Insights into a challenging symptom. J. Neurol. Sci..

[44] Negrini F., Ferrario I., Mazziotti D., Berchicci M., Bonazzi M., de Sire A., Negrini S., Zapparoli L. (2021). Neuropsychological Features of Severe Hospitalized Coronavirus Disease 2019 Patients at Clinical Stability and Clues for Postacute Rehabilitation. Arch. Phys. Med. Rehabil..

[45] Versace V., Sebastianelli L., Ferrazzoli D., Romanello R., Ortelli P., Saltuari L., D’Acunto A., Porrazzini F., Ajello V., Oliviero A. (2021). Intracortical GABAergic dysfunction in patients with fatigue and dysexecutive syndrome after COVID-19. Clin. Neurophysiol..

[46] Yesilkaya U.H., Sen M., Balcioglu Y.H. (2021). COVID-19-related cognitive dysfunction may be associated with transient disruption in the DLPFC glutamatergic pathway. J. Clin. Neurosci..

[47] Beaud V., Crottaz-Herbette S., Dunet V., Vaucher J., Bernard-Valnet R., Du Pasquier R., Bart P.-A., Clarke S. (2021). Pattern of cognitive deficits in severe COVID-19. J. Neurol. Neurosurg. Psychiatry.

[48] Gautam N., Madathil S., Tahani N., Bolton S., Parekh D., Stockley J., Goyal S., Qureshi H., Yasmin S., Cooper B.G. (2022). Medium-Term Outcomes in Severely to Critically Ill Patients With Severe Acute Respiratory Syndrome Coronavirus 2 Infection. Clin. Infect. Dis..

[49] Groiss S.J., Balloff C., Elben S., Brandenburger T., Müttel T., Kindgen-Milles D., Vollmer C., Feldt T., Kunstein A., Ole Jensen B.-E. (2020). Prolonged Neuropsychological Deficits, Central Nervous System Involvement, and Brain Stem Affection After COVID-19—A Case Series. Front. Neurol..

[50] Hellmuth J., Barnett T.A., Asken B.M., Kelly J.D., Torres L., Stephens M.L., Greenhouse B., Martin J.N., Chow F.C., Deeks S.G. (2021). Persistent COVID-19-associated neurocognitive symptoms in non-hospitalized patients. J. NeuroVirol..

[51] Tolentino J.C., Gjorup A.L.T., Schmidt G.J., Schmidt S.L. (2021). Early attention impairment in a patient with COVID-19. Psychiatry Clin. Neurosci..

[52] Whiteside D.M., Oleynick V., Holker E., Waldron E.J., Porter J., Kasprzak M. (2021). Neurocognitive deficits in severe COVID-19 infection: Case series and proposed model. Clin. Neuropsychol..

[53] Vannorsdall T.D., Brigham E., Fawzy A., Raju S., Gorgone A., Pletnikova A., Lyketsos C.G., Parker A.M., Oh E.S. (2022). Cognitive Dysfunction, Psychiatric Distress, and Functional Decline After COVID-19. J. Acad. Consult. Liaison Psychiatry.

[54] Henneghan A.M., Lewis K.A., Gill E., Kesler S.R. (2022). Cognitive Impairment in Non-critical, Mild-to-Moderate COVID-19 Survivors. Front. Psychol..

[55] Graham E.L., Clark J.R., Orban Z.S., Lim P.H., Szymanski A.L., Taylor C., DiBiase R.M., Jia D.T., Balabanov R., Ho S.U. (2021). Persistent neurologic symptoms and cognitive dysfunction in non-hospitalized Covid-19 “long haulers”. Ann. Clin. Transl. Neurol..

[56] Del Brutto O.H., Wu S., Mera R.M., Costa A.F., Recalde B.Y., Issa N.P. (2021). Cognitive decline among individuals with history of mild symptomatic SARS-CoV-2 infection: A longitudinal prospective study nested to a population cohort. Eur. J. Neurol..

[57] Abdelghani M., Atwa S.A., Said A., Zayed N.E., Abdelmoaty A.A., Hassan M.S. (2022). Cognitive after-effects and associated correlates among post-illness COVID-19 survivors: A cross-sectional study, Egypt. Egypt. J. Neurol. Psychiatry Neurosurg..

[58] Miskowiak K.W., Fugledalen L., Jespersen A.E., Sattler S.M., Podlekareva D., Rungby J., Porsberg C.M., Johnsen S. (2022). Trajectory of cognitive impairments over 1 year after COVID-19 hospitalisation: Pattern, severity, and functional implications. Eur. Neuropsychopharmacol..

[59] Rubega M., Ciringione L., Bertuccelli M., Paramento M., Sparacino G., Vianello A., Masiero S., Vallesi A., Formaggio E., Del Felice A. (2022). High-density EEG sleep correlates of cognitive and affective impairment at 12-month follow-up after COVID-19. Clin. Neurophysiol..

[60] Vialatte de Pémille C., Ray A., Michel A., Stefano F., Yim T., Bruel C., Zuber M. (2022). Prevalence and prospective evaluation of cognitive dysfunctions after SARS due to SARS-CoV-2 virus. The COgnitiVID study. Rev. Neurol..

[61] Almeria M., Cejudo J.C., Sotoca J., Deus J., Krupinski J. (2020). Cognitive profile following COVID-19 infection: Clinical predictors leading to neuropsychological impairment. Brain Behav. Immun. Health.

[62] Cian V., De Laurenzis A., Siri C., Gusmeroli A., Canesi M. (2022). Cognitive and Neuropsychiatric Features of COVID-19 Patients After Hospital Dismission: An Italian Sample. Front. Psychol..

[63] Cecchetti G., Agosta F., Canu E., Basaia S., Barbieri A., Cardamone R., Bernasconi M.P., Castelnovo V., Cividini C., Cursi M. (2022). Cognitive, EEG, and MRI features of COVID-19 survivors: A 10-month study. J. Neurol..

[64] Johnsen S., Sattler S.M., Miskowiak K.W., Kunalan K., Victor A., Pedersen L., Andreassen H.F., Jørgensen B.J., Heebøll H., Andersen M.B. (2021). Descriptive analysis of long COVID sequelae identified in a multidisciplinary clinic serving hospitalised and non-hospitalised patients. ERJ Open Res..

[65] Zhou H., Lu S., Chen J., Wei N., Wang D., Lyu H., Shi C., Hu S. (2020). The landscape of cognitive function in recovered COVID-19 patients. J. Psychiatr. Res..

[66] Aiello E.N., Radici A., Mora G., Pain D. (2022). Cognitive phenotyping of post-infectious SARS-CoV-2 patients. Neurol. Sci..

[67] Frontera J.A., Yang D., Lewis A., Patel P., Medicherla C., Arena V., Fang T., Andino A., Snyder T., Madhavan M. (2021). A prospective study of long-term outcomes among hospitalized COVID-19 patients with and without neurological complications. J. Neurol. Sci..

[68] Manera M.R., Fiabane E., Pain D., Aiello E.N., Radici A., Ottonello M., Padovani M., Wilson B.A., Fish J., Pistarini C. (2022). Clinical features and cognitive sequelae in COVID-19: A retrospective study on N=152 patients. Neurol. Sci..

[69] Serrano-Castro P.J., Garzón-Maldonado F.J., Casado-Naranjo I., Ollero-Ortiz A., Mínguez-Castellanos A., Iglesias-Espinosa M., Baena-Palomino P., Sánchez-Sanchez V., Sánchez-Pérez R.M., Rubi-Callejon J. (2022). The cognitive and psychiatric subacute impairment in severe Covid-19. Sci. Rep..

[70] Amalakanti S., Arepalli K.V.R., Jillella J.P. (2021). Cognitive assessment in asymptomatic COVID-19 subjects. VirusDisease.

[71] Guo P., Benito Ballesteros A., Yeung S.P., Liu R., Saha A., Curtis L., Kaser M., Haggard M.P., Cheke L.G. (2022). COVCOG 2: Cognitive and Memory Deficits in Long COVID: A Second Publication From the COVID and Cognition Study. Front. Aging Neurosci..

[72] Holdsworth D.A., Chamley R., Barker-Davies R., O’Sullivan O., Ladlow P., Mitchell J.L., Dewson D., Mills D., May S.L.J., Cranley M. (2022). Comprehensive clinical assessment identifies specific neurocognitive deficits in working-age patients with long-COVID. PLoS ONE.

[73] Mattioli F., Stampatori C., Righetti F., Sala E., Tomasi C., De Palma G. (2021). Neurological and cognitive sequelae of Covid-19: A four month follow-up. J. Neurol..

[74] Raman B., Cassar M.P., Tunnicliffe E.M., Filippini N., Griffanti L., Alfaro-Almagro F., Okell T., Sheerin F., Xie C., Mahmod M. (2021). Medium-term effects of SARS-CoV-2 infection on multiple vital organs, exercise capacity, cognition, quality of life and mental health, post-hospital discharge. EClinicalMedicine.

[75] Ermis U., Rust M.I., Bungenberg J., Costa A., Dreher M., Balfanz P., Marx G., Wiesmann M., Reetz K., Tauber S.C. (2021). Neurological symptoms in COVID-19: A cross-sectional monocentric study of hospitalized patients. Neurol. Res. Pract..

[76] Jaywant A., Vanderlind W.M., Alexopoulos G.S., Fridman C.B., Perlis R.H., Gunning F.M. (2021). Frequency and profile of objective cognitive deficits in hospitalized patients recovering from COVID-19. Neuropsychopharmacology.

[77] Patel R., Savrides I., Cahalan C., Doulatani G., O’Dell M.W., Toglia J., Jaywant A. (2021). Cognitive impairment and functional change in COVID-19 patients undergoing inpatient rehabilitation. Int. J. Rehabil. Res..

[78] Albu S., Zozaya N.R., Murillo N., García-Molina A., Chacón C.A.F., Kumru H. (2021). What’s going on following acute covid-19? Clinical characteristics of patients in an out-patient rehabilitation program. NeuroRehabilitation.

[79] Bolattürk Ö.F., Soylu A.C. (2022). Evaluation of cognitive, mental, and sleep patterns of post-acute COVID-19 patients and their correlation with thorax CT. Acta Neurol. Belg..

[80] De Lorenzo R., Conte C., Lanzani C., Benedetti F., Roveri L., Mazza M.G., Brioni E., Giacalone G., Canti V., Sofia V. (2020). Residual clinical damage after COVID-19: A retrospective and prospective observational cohort study. PLoS ONE.

[81] Dressing A., Bormann T., Blazhenets G., Schroeter N., Walter L.I., Thurow J., August D., Hilger H., Stete K., Gerstacker K. (2022). Neuropsychologic Profiles and Cerebral Glucose Metabolism in Neurocognitive Long COVID Syndrome. J. Nucl. Med..

[82] Evans R.A., McAuley H., Harrison E.M., Shikotra A., Singapuri A., Sereno M., Elneima O., Docherty A.B., Lone N.I., Leavy O.C. (2021). Physical, cognitive, and mental health impacts of COVID-19 after hospitalisation (PHOSP-COVID): A UK multicentre, prospective cohort study. Lancet Respir. Med..

[83] Ferrucci R., Dini M., Groppo E., Rosci C., Reitano M.R., Bai F., Poletti B., Brugnera A., Silani V., D’Arminio Monforte A. (2021). Long-Lasting Cognitive Abnormalities after COVID-19. Brain Sci..

[84] García-Sánchez C., Calabria M., Grunden N., Pons C., Arroyo J.A., Gómez-Anson B., Lleó A., Alcolea D., Belvís R., Morollón N. (2022). Neuropsychological deficits in patients with cognitive complaints after COVID-19. Brain Behav..

[85] Hadad R., Khoury J., Stanger C., Fisher T., Schneer S., Ben-Hayun R., Possin K., Valcour V., Aharon-Peretz J., Adir Y. (2022). Cognitive dysfunction following COVID-19 infection. J. Neurovirol..

[86] Hellgren L., Birberg Thornberg U., Samuelsson K., Levi R., Divanoglou A., Blystad I. (2021). Brain MRI and neuropsychological findings at long-term follow-up after COVID-19 hospitalisation: An observational cohort study. BMJ Open.

[87] Leth S., Gunst J.D., Mathiasen V., Hansen K., Søgaard O., Østergaard L., Jensen-Fangel S., Storgaard M., Agergaard J. (2021). Persistent Symptoms in Patients Recovering From COVID-19 in Denmark. Open Forum Infect. Dis..

[88] Mazza M.G., Palladini M., De Lorenzo R., Magnaghi C., Poletti S., Furlan R., Ciceri F., Rovere-Querini P., Benedetti F. (2021). Persistent psychopathology and neurocognitive impairment in COVID-19 survivors: Effect of inflammatory biomarkers at three-month follow-up. Brain Behav. Immun..

[89] Méndez R., Balanzá-Martínez V., Luperdi S.C., Estrada I., Latorre A., González-Jiménez P., Feced L., Bouzas L., Yépez K., Ferrando A. (2021). Short-term neuropsychiatric outcomes and quality of life in COVID-19 survivors. J. Intern. Med..

[90] Monti G., Leggieri C., Fominskiy E., Scandroglio A.M., Colombo S., Tozzi M., Moizo E., Mucci M., Crivellari M., Pieri M. (2021). Two-months quality of life of COVID-19 invasively ventilated survivors; an Italian single-center study. Acta Anaesthesiol. Scand..

[91] Puchner B., Sahanic S., Kirchmair R., Pizzini A., Sonnweber B., Wöll E., Mühlbacher A., Garimorth K., Dareb B., Ehling R. (2021). Beneficial effects of multi-disciplinary rehabilitation in postacute COVID-19: An observational cohort study. Eur. J. Phys. Rehabil. Med..

[92] Rass V., Beer R., Schiefecker A.J., Kofler M., Lindner A., Mahlknecht P., Heim B., Limmert V., Sahanic S., Pizzini A. (2021). Neurological outcome and quality of life 3 months after COVID-19: A prospective observational cohort study. Eur. J. Neurol..

[93] Soldati A.B., Almeida C., Lima M., Araujo A., Araujo-Leite M.A., Silva M.T.T. (2021). Telephone Screening of Cognitive Status (TICS) in severe COVID-19 patients: Utility in the era of social isolation. eNeurologicalSci.

[94] van den Borst B., Peters J.B., Brink M., Schoon Y., Bleeker-Rovers C.P., Schers H., van Hees H.W.H., van Helvoort H., van den Boogaard M., van der Hoeven H. (2021). Comprehensive Health Assessment 3 Months After Recovery From Acute Coronavirus Disease 2019 (COVID-19). Clin. Infect. Dis..

[95] Group T.W.C.f.t.C.S. (2021). Four-Month Clinical Status of a Cohort of Patients After Hospitalization for COVID-19. JAMA.

[96] Hosp J.A., Dressing A., Blazhenets G., Bormann T., Rau A., Schwabenland M., Thurow J., Wagner D., Waller C., Niesen W.D. (2021). Cognitive impairment and altered cerebral glucose metabolism in the subacute stage of COVID-19. Brain.

[97] Latronico N., Peli E., Calza S., Rodella F., Novelli M.P., Cella A., Marshall J., Needham D.M., Rasulo F.A., Piva S. (2022). Physical, cognitive and mental health outcomes in 1-year survivors of COVID-19-associated ARDS. Thorax.

[98] Venturelli S., Benatti S.V., Casati M., Binda F., Zuglian G., Imeri G., Conti C., Biffi A.M., Spada M.S., Bondi E. (2021). Surviving COVID-19 in Bergamo province: A post-acute outpatient re-evaluation. Epidemiol. Infect..

[99] Weihe S., Mortensen C.B., Haase N., Andersen L.P.K., Mohr T., Siegel H., Ibsen M., Jørgensen V.R.L., Buck D.L., Pedersen H.B.S. (2022). Long-term cognitive and functional status in Danish ICU patients with COVID-19. Acta Anaesthesiol. Scand..

[100] Becker J.H., Lin J.J., Doernberg M., Stone K., Navis A., Festa J.R., Wisnivesky J.P. (2021). Assessment of Cognitive Function in Patients After COVID-19 Infection. JAMA Netw. Open.

[101] Bonizzato S., Ghiggia A., Ferraro F., Galante E. (2022). Cognitive, behavioral, and psychological manifestations of COVID-19 in post-acute rehabilitation setting: Preliminary data of an observational study. Neurol. Sci..

[102] Walle-Hansen M.M., Ranhoff A.H., Mellingsæter M., Wang-Hansen M.S., Myrstad M. (2021). Health-related quality of life, functional decline, and long-term mortality in older patients following hospitalisation due to COVID-19. BMC Geriatr..

[103] Sardella A., Chiara E., Alibrandi A., Bellone F., Catalano A., Lenzo V., Quattropani M.C., Basile G. (2022). Changes in Cognitive and Functional Status and in Quality of Life of Older Outpatients during the COVID-19 Pandemic. Gerontology.

[104] Buonsenso D., Munblit D., De Rose C., Sinatti D., Ricchiuto A., Carfi A., Valentini P. (2021). Preliminary evidence on long COVID in children. Acta Paediatr..

[105] Giordano G.M., Brando F., Pezzella P., De Angelis M., Mucci A., Galderisi S. (2022). Factors influencing the outcome of integrated therapy approach in schizophrenia: A narrative review of the literature. Front. Psychiatry.

[106] Giuliani L., Giordano G.M., Bucci P., Pezzella P., Brando F., Galderisi S. (2021). Improving Knowledge on Pathways to Functional Outcome in Schizophrenia: Main Results From the Italian Network for Research on Psychoses. Front. Psychiatry.

[107] Penninx B. (2021). Psychiatric symptoms and cognitive impairment in “Long COVID”: The relevance of immunopsychiatry. World Psychiatry.

[108] Greenberg N., Rafferty L. (2021). Post-traumatic stress disorder in the aftermath of COVID-19 pandemic. World Psychiatry.

[109] Holt-Lunstad J. (2021). A pandemic of social isolation?. World Psychiatry.

[110] Wang Q., Xu R., Volkow N.D. (2021). Increased risk of COVID-19 infection and mortality in people with mental disorders: Analysis from electronic health records in the United States. World Psychiatry.

[111] Stewart D.E., Appelbaum P.S. (2020). COVID-19 and psychiatrists’ responsibilities: A WPA position paper. World Psychiatry.

[112] Pinto T.C.C., Machado L., Bulgacov T.M., Rodrigues-Júnior A.L., Costa M.L.G., Ximenes R.C.C., Sougey E.B. (2019). Is the Montreal Cognitive Assessment (MoCA) screening superior to the Mini-Mental State Examination (MMSE) in the detection of mild cognitive impairment (MCI) and Alzheimer’s Disease (AD) in the elderly?. Int. Psychogeriatr..

[113] Tsoi K.K., Chan J.Y., Hirai H.W., Wong S.Y., Kwok T.C. (2015). Cognitive Tests to Detect Dementia: A Systematic Review and Meta-analysis. JAMA Intern. Med..

[114] Aboujaoude E., Gega L., Saltarelli A.J. (2021). The retention challenge in remote therapy and learning seen through the lens of the COVID-19 pandemic. World Psychiatry.

[115] Maleki Dana P., Sadoughi F., Hallajzadeh J., Asemi Z., Mansournia M.A., Yousefi B., Momen-Heravi M. (2020). An Insight into the Sex Differences in COVID-19 Patients: What are the Possible Causes?. Prehosp. Disaster Med..

[116] Michelutti M., Furlanis G., Buoite Stella A., Bellavita G., Frezza N., Torresin G., Ajčević M., Manganotti P. (2022). Sex-dependent characteristics of Neuro-Long-COVID: Data from a dedicated neurology ambulatory service. J. Neurol. Sci..

[117] Bai F., Tomasoni D., Falcinella C., Barbanotti D., Castoldi R., Mulè G., Augello M., Mondatore D., Allegrini M., Cona A. (2022). Female gender is associated with long COVID syndrome: A prospective cohort study. Clin. Microbiol. Infect..

[118] Pletzer B., Harris T.-A., Scheuringer A., Hidalgo-Lopez E. (2019). The cycling brain: Menstrual cycle related fluctuations in hippocampal and fronto-striatal activation and connectivity during cognitive tasks. Neuropsychopharmacology.

[119] Eshkoor S.A., Hamid T.A., Mun C.Y., Ng C.K. (2015). Mild cognitive impairment and its management in older people. Clin. Interv. Aging.

[120] Reynolds C.F., Jeste D.V., Sachdev P.S., Blazer D.G. (2022). Mental health care for older adults: Recent advances and new directions in clinical practice and research. World Psychiatry.

[121] Solomon T. (2021). Neurological infection with SARS-CoV-2—The story so far. Nat. Rev. Neurol..

[122] Biagianti B., Di Liberto A., Nicolò Edoardo A., Lisi I., Nobilia L., de Ferrabonc G.D., Zanier E.R., Stocchetti N., Brambilla P. (2022). Cognitive Assessment in SARS-CoV-2 Patients: A Systematic Review. Front. Aging Neurosci..

[123] Sasannejad C., Ely E.W., Lahiri S. (2019). Long-term cognitive impairment after acute respiratory distress syndrome: A review of clinical impact and pathophysiological mechanisms. Crit. Care.

[124] Bandala C., Cortes-Altamirano J.L., Reyes-Long S., Lara-Padilla E., Ilizaliturri-Flores I., Alfaro-Rodríguez A. (2021). Putative mechanism of neurological damage in COVID-19 infection. Acta Neurobiol. Exp..

[125] Hawkins M., Sockalingam S., Bonato S., Rajaratnam T., Ravindran M., Gosse P., Sheehan K.A. (2021). A rapid review of the pathoetiology, presentation, and management of delirium in adults with COVID-19. J. Psychosom. Res..

[126] Grasby K.L., Jahanshad N., Painter J.N., Colodro-Conde L., Bralten J., Hibar D.P., Lind P.A., Pizzagalli F., Ching C.R.K., McMahon M.A.B. (2020). The genetic architecture of the human cerebral cortex. Science.

[127] Areza-Fegyveres R., Kairalla R.A., Carvalho C.R.R., Nitrini R. (2010). Cognition and chronic hypoxia in pulmonary diseases. Dement. Neuropsychol..

[128] Menon V. (2020). Brain networks and cognitive impairment in psychiatric disorders. World Psychiatry.

[129] Alemanno F., Houdayer E., Parma A., Spina A., Del Forno A., Scatolini A., Angelone S., Brugliera L., Tettamanti A., Beretta L. (2021). COVID-19 cognitive deficits after respiratory assistance in the subacute phase: A COVID-rehabilitation unit experience. PLoS ONE.

[130] Vanderlind W.M., Rabinovitz B.B., Miao I.Y., Oberlin L.E., Bueno-Castellano C., Fridman C., Jaywant A., Kanellopoulos D. (2021). A systematic review of neuropsychological and psychiatric sequalae of COVID-19: Implications for treatment. Curr. Opin. Psychiatry.

[131] Altuna M., Sánchez-Saudinós M.B., Lleó A. (2021). Cognitive symptoms after COVID-19. Neurol. Perspect..

[132] Daroische R., Hemminghyth M.S., Eilertsen T.H., Breitve M.H., Chwiszczuk L.J. (2021). Cognitive Impairment After COVID-19—A Review on Objective Test Data. Front. Neurol..

[133] Rabinovitz B., Jaywant A., Fridman C.B. (2020). Neuropsychological functioning in severe acute respiratory disorders caused by the coronavirus: Implications for the current COVID-19 pandemic. Clin. Neuropsychol..

