# Supplementary material for: Cognitive Impairment after Post-Acute COVID-19 Infection: A Systematic Review of the Literature
Source: J Pers Med. 2022 Dec 15;12(12):2070. doi: 10.3390/jpm12122070 (PMC9781311; doi:10.3390/jpm12122070)
Supplement: Supplementary file 1 [file jpm-12-02070-s001.zip › jpm-2068407-supplementary.pdf]

Index

- **Table S1:** Main characteristics of the articles included
- **References**

Supplementary Table S1: Main characteristics of the articles included (n=72)

Author(s), Year, Study Design	Sample size or Number of Included Studies	Cognitive assessment scales	Other assessments	Main results	Limitations of the study
Ceban et al. (2022) [1]<br><br>Systematic review and Meta-Analysis	N = 81 studies, 43 of which evaluating cognitive impairment	- MoCA<br>- BRB-NT<br>- OMC<br>- MMSE<br>- BACS	- Laboratory testing (inflammatory parameters)	- Subjects with cognitive impairment = 22%<br>- No statistically significant differences in cognitive impairment between female and males or between hospitalized and non-hospitalized patients<br>- 9 of 14 studies reported the presence of both proinflammatory markers and cognitive impairment	- Lack of pre-COVID cognitive assessments<br>- Samples not stratified by disease severity<br>- Findings may not directly result from the infection<br>- Recruitment bias<br>- Use of cognitive screening tools and measures of general cognitive functioning<br>-
Crivelli et al. (2022) [2]<br><br>Systematic review and Meta-Analysis	N = 32 studies<br><br>N of subjects = 2103 patients (56% M)<br><br>N of healthy controls = 506 (50% M)	- MoCA<br>- MMSE<br>- FAB<br>- TICS-M<br>- TMT<br>- SCT<br>- CPT<br>- Digit span<br>- RAVLT<br>- BVMT-R<br>- CVLT<br>- SCID-D<br>- Stroop	- BDI<br>- PHQ-9<br>- GAD-7<br>- Cerebral FDG-PET	- Significantly lower scores in cognition in the post-COVID-19 patient group compared to controls<br>- Deficits in global scores of screening measures and sub-scores of attention, memory, and executive functions<br>- Meta-analysis reported an effect of COVID-19 infection on the total MoCA score (MD=−0.94, 95% CI −1.59, −0.29; P = .0049)<br>- Meta-regression analysis reported that an increase in age correlates with enhanced cognitive dysfunction	- Some studies designed with small sample sizes<br>- Use of cognitive screening tools and measures of general cognitive functioning<br>- Findings may not directly result from the infection<br>- Heterogeneity of the outcome of the assessments<br>- Heterogeneous samples<br>- Lack of long-term follow-up
Tavares-Junior et al. (2022) [3]<br><br>Systematic review	N = 22 studies	- MoCA	- RMN<br>- PET	- Cognitive impairment varied from 2.6% to 81% within the samples considered.<br>- The studies did not find specific alterations in structural neuroimaging exams, except two studies that found	- Lack of pre-COVID cognitive assessment<br>- Methodological differences between the study designs

				frontoparietal hypometabolism in patients with encephalopathy	- Lack of control groups in some studies
Schou et al. (2021) [4]<br><br>Systematic review	N = 66 studies	- MMSE<br>- MoCA<br>- CogState<br>- PROMIS<br>- TMT<br>- SCT<br>- CPT<br>- Digit span<br>- RDS	- MRI scans at the 3-month follow-up<br>- CRP<br>- LDH<br>- CFQ<br>- STAI<br>- BDI	- 11 studies reported cognitive deficits in >25% of their patient populations<br>- Deficits in concentration problems, memory, attention, language, praxis abilities, encoding and verbal fluency<br>- MRI scans showed that impaired patients displayed higher bilateral grey matter volume loss in the hippocampus	- Lack of control groups<br>- Heterogeneous sample sizes<br>- Study instruments not always appropriate
Vanderlind et al. (2021) [5]<br><br>Systematic review	N = 33 studies	- MoCA<br>- MMSE<br>- TICS-M	- MRI	- 15.0–40.0% of participants presented cognitive impairment 10–105 days after hospital discharge<br>- The most affected domains were sustained attention, executive function attention, memory and language<br>- Subjects treated with oxygen therapy had lower scores in the domains of memory, attention, working memory, processing speed, executive function, and global cognition	- Limited time frame of evaluations<br>- Sample not stratified by disease severity
Altuna et al. (2021) [6]<br><br>Narrative review	N =154 studies	- MoCA<br>- MMSE<br>- CPT	- Laboratory testing (inflammatory parameters)<br>- MRI<br>- Brain FDG-PET	- Cognitive sequelae are frequent after COVID-19, even in mild cases not requiring hospitalization or ICU admission<br>- Most affected domain was executive function<br>- Frontoparietal hypometabolism correlated with MoCA performance	- Use of cognitive screening tools and measures of general cognitive functioning
Daroische et al. (2021) [7]<br><br>Review	N = 12 studies	- MoCA<br>- MMSE<br>- TICS<br>- TMT-A<br>- FAB<br>- Tests of memory	- NA	- The percentage of patients with global cognitive impairment ranged from 15% to 80%	- Small number of studies

				- Impairment on attention and executive functions<br>- Some studies reported memory difficulties, with two studies reporting short-term memory deficits	
Rabnovitz et al. (2020) [8]<br><br>Review	N =14 studies	- MoCA<br>- Brief Memory and Executive Test<br>- Dyscontrol Scale<br>- Repeatable Battery for the Assessment of Neuropsychological Status<br>- Weekly Calendar Planning Activity<br>- Executive Function Performance Test<br>- Kettle Test	- HADS<br>- Geriatric Depression Scale-short form	- Survivors of COVID-19 who are extubated appear to be experiencing high rates of cognitive impairment, anxiety, and mood symptoms.<br>- Most patients exhibited a dysexecutive syndrome consisting of inattention, disorientation, and difficulties organizing response to command	- Heterogeneous sample (not stratified by disease severity)<br>- Lack of pre-COVID cognitive assessments<br>- Use of cognitive screening tools and measures of general cognitive functioning
Weihe et al. (2022) [9]<br><br>Prospective cohort	N= 105 patients (100% hospitalized in ICU; median age 67y; 70% M)	- MiniMoCA (telephone interview)	- EQ-5D-5L<br>- ADL<br>- IADL<br>- FAS<br>- CFS	- 26% (n=27) had cognitive scores indicating impaired cognitive function (MiniMoCA <11) at 6 months, and 17% (n=16) at 12 months.<br>- No association was found between cognitive function and time on ventilator.	- Small sample size<br>- Large number of dropouts<br>- Lack of pre-COVID cognitive assessments
Bonizzato et al. (2021) [10]<br>Prospective cohort study	N=12 (mean age 71.33y; 58,3 % M)	- MMSE<br>- MoCA<br>- Digit span forward and backwards<br>- RAVL<br>- SPART<br>- SDMT<br>- TMT<br>- Stroop Test<br>- FAB<br>- Fonemic Fluency FAS	- NPI<br>- AD-R	-Number of patients with test scores below the threshold values:<br>- MMSE:<br>o T0(58, 3%- 7/12)<br>o T1(33,3%- 4/12)<br>o T2(25%- 2/8)<br>- MoCA:<br>o T0(50%- 6/12)<br>o T1(50%- 6/12)<br>o T2(50%- 4/8)<br>No significant differences were found over time in MMSE and MoCA total scores	- Small sample size<br>- Large number of dropouts<br>- Heterogenous sample (not stratified by age)
Holdsworth et al. (2022) [11]	N= 205 (mean age 39y; 83,4%	- NIH-TB	- FAS<br>- GAD-7	- The fluid composite scores were lower than crystallized composite	- Control sample not matched by age and weight

Prospective cohort	M; 100% hospitalized)<br>N of controls = 146630 (mean age 31.8y; 89% M)		- PHQ-9	scores by a mean difference in T-score of 4.7 (p<0.001).<br>- Cognitive scores did not differ significantly between community and hospitalized patients	- Use of cognitive screening tools and measures of general cognitive functioning
Rubega et al. (2022) [12]<br><br>Prospective cohort	N= 33 patients (73% M, 100% hospitalized)<br><br>N of controls = 12 (67% M)	- MoCA<br>- FAB<br>- Stroop task<br>- Digit Span forward and backward<br>- RAVLT<br>- SDMT<br>- TMT	- BDI<br>- PTSD<br>- PCS-12 and MCS-12<br>- PSQI<br>- EEG	- Trend towards worse performance in executive functions in patients, in particular in non-ICU patients<br>- Higher likelihood of PTSD correlated to a worse performance in Digit span backward and TMT-B<br>- A higher score in BDI is correlated to a lower score in MoCA<br>- Multiple linear regression analyses highlighted that non-ICU patients got lower scores in cognitive tasks evaluating executive function and working memory	- Small sample size<br>- Lack of face-to-face cognitive evaluation
Vialatte de Pémille et al. (2022) [13]<br><br>Prospective cohort	N= 13 (mean age 62 y; 61,5% M;100% hospitalized in ICU)	- MMSE<br>- FAB<br>- 40 Words oral naming test<br>- Dubois five words test<br>- Digit span forward and backward<br>- Similarities test of the WAIS IV<br>- Brixton test<br>- Stroop Color - Word Test - Victoria version<br>- Categorical and lexical verbal fluencies<br>- Common bedside praxis	- MADRS	- A total of 92% patients exhibited abnormal global cognitive function according to the MMSE score and 46% had space and temporal disorientation.<br>- Significant differences between baseline and follow-up evaluations were observed for two of the five global tests: MMSE and FAB test	- Small sample size<br>- Lack of brain imaging evaluations
García-Sánchez et al. (2022) [14]<br><br>Prospective cohort	N= 63 (mean age 51.1y; 35% M; 52,4% hospitalized)	- MoCA<br>- CPT-II<br>- RAVLT<br>- ROCFT<br>- BNT<br>- Digit Span Forward and Backward<br>- Block Design<br>- Coding test	- CRP levels<br>- AST<br>- ALT<br>- LDH<br>- CK<br>- Hemoglobin<br>- Platelets<br>- Leukocytes<br>- Lymphocyte<br>- D-dimer	- Multiple-domain impairment (60.3%) was more frequent than impairment in only one domain (39.7%)<br>- Attention deficits were the most frequent types of deficits in patients with single domain impairment	- Lack of control group<br>-

		- Symbol Search<br>- TMT<br>- Stroop verbal fluency tasks<br>- 15-Objects Test	- Ferritin<br>- IL-6		
Vannorsdall et al. (2022) [15]<br><br>Prospective cohort	N=82 (mean age 54.5y; 34% M; 100 % hospitalized)	- RAVLT<br>- Oral TMT-A and B<br>- Digit span forward and backward<br>- Letter-cued verbal fluency<br>- Category-cued verbal fluency	- PHQ-9<br>- GAD-7<br>- IES-6<br>- QDRS	- Post-ICU clinic patients produced lower cognitive composite scores than non-ICU patients<br>• Mean post-ICU = 90.6 (SD = 11.0)<br>• Mean non-ICU = 95.8 (SD = 10.3)<br>- Non-ICU patients= 0.28 standard deviations below demographic expectation<br>- Post-ICU patients= 0.63 standard deviations below expectation	- Sample may not be representative of the entire affected population<br>- Lack of pre-COVID cognitive assessment<br>- Lack of control group<br>- Methodological bias (those who could not complete tasks were excluded)<br>- Lack of follow-up
Frontera et al. (2021) [16]<br><br>Prospective cohort	N = 395 patients<br><br>N of controls= 395 patients<br><br>Both= mean age: 68.0y; 65% M, 100% hospitalized)	- MoCA (telephone interview)	- NeuroQol	- Both groups of patients had high rates of cognitive impairment= 50% at 6-months.<br>- In the cohort with neurological complications, 50 had impaired cognition	- Methodological bias (control group was equally affected by COVID-19)<br>- Findings may not directly result from the infection<br>- Use of cognitive screening tools and measures of general cognitive functioning
Evans et al. (2021) [17]<br><br>Prospective cohort	N = 1077 patients (mean age 57.9 y; 64,3% M;100% hospitalized)	- MoCA (888 patients)	- BNP<br>- NT-BNP<br>- eGFR<br>- HbA1C<br>- D-dimer<br>- CRP<br>- EQ-5D-5L<br>- PHQ-9<br>- GAD-7	- 16,9% of patients showed a MoCA score < 23<br>- The severity of physical and mental health impairments was closely related, whereas cognitive health impairments were independent<br>- Age had a non-linear association, with age groups <30 years and >70 years perceiving better recovery than those aged 50–59 years	- Sample may not be representative of the entire affected population

Miskowiak (2021) [18]<br><br>Prospective cohort	N =29 patients (mean age 56.2; 59% M; 100% hospitalized)	- SCIP-D<br>- TMT- B	- Biomarkers of inflammation<br>- WPAI<br>- EQ-5D-5L	- 65% suffer from clinically relevant cognitive impairments (most affected: verbal learning and executive function; moderate impairments: working memory, verbal fluency and psychomotor speed.<br>- Higher maximum d-dimer levels correlated with poorer verbal recall and psychomotor speed.<br>- Poorer verbal memory and lower psychomotor speed correlated with higher d-dimer levels	- Small sample size<br>- Lack of control group<br>- Methodological bias (cross-sectional design)<br>- Use of cognitive screening tools and measures of general cognitive functioning
Graham et al. (2021) [19]<br><br>Prospective cohort	N = 100 (mean age 43.2y; 30% M; Non hospitalized)	- NIH-TB v2.1 (36% of the cohort: 48% COVID+/ 24% COVID-)	- Markers of inflammation<br>- PROMIS quality of life (subjective)<br>- Brain MRI<br>- Spine MRI<br>- EEG<br>- EMG	- PROMIS and NIH Toolbox results were not significantly different between patients and controls<br>- SARS-CoV-2+ patients had significantly worse NIH Toolbox cognitive function in attention and working memory domains.<br>- Both patients and controls had significantly worse than expected PROMIS quality of life for cognition<br>- No difference between the two groups was found at imaging	- Small sample size<br>- Sample may not be representative of the entire affected population<br>- Lack of face-to-face cognitive evaluation<br>- Lack of pre-COVID cognitive assessments<br>- Lack of follow-up<br>- Methodological bias (not every patient had the same set of laboratory, imaging, and neurophysiologic testing)
Mattioli et al. (2021) [20]<br><br>Prospective cohort	N = 120 patients (mean age 47.86y; 25% M)<br><br>N of controls= 30 (mean age 45.73y; 26.7% M)	- COWA<br>- RCFT<br>- CVLT<br>- TEA attention test<br>- Tower of London<br>- MMSE	- DASS-21	- The mean number of impaired neuropsychological tests was 1.69 in COVID-19 and 1 in non-COVID-19 subjects (not statistically significant)<br>- Mean scores of all the neuropsychological tests were not statistically different	- Sample not stratified by disease severity<br>- Lack of pre-COVID cognitive assessments
Hosp et al. (2021) [21]<br><br>Prospective cohort	N = 29 (mean age 65,2y; 62% M; 100% hospitalized)	- MoCA<br>- HVLT-R<br>- Digit span forward and reverse	- Brain MRI<br>- 18-FDG PET imaging	- Impaired performance on the MoCA in 18/26 patients (3 did not complete evaluations)	- Selection bias (only younger people accepted the evaluations)

		- SDMT<br>- TMT-A and B<br>- Semantic fluency test<br>- Phonemic fluency test<br>- Stroop test		○ 54% were mild to moderate impaired<br>○ 15% were severely impaired<br>○ The most affected domains were executive abilities, visuoconstruction, memory and attention<br>- 13/15 patients had low scores in the extensive battery<br>○ Memory and executive functions were the most affected domains<br>- There was a highly significant linear relationship between cognitive assessment and PET (a higher pattern expression score was associated with worse cognitive performance)	- Sample may not be representative of the entire affected population
Leth et al. (2021) [22]<br><br>Prospective cohort	N = 49 (mean age 58 y; 43% M; 100% hospitalized)	-OMCTest	NA	- 39% after 6 weeks and 45% after 12 weeks reported concentration difficulties<br>- 21% after 6 weeks and 11% after 12 weeks showed impaired OMC test	- Small sample size<br>- Single-center study<br>- Lack of control group.<br>- Lack of objective measurements<br>- High rates of loss to follow-up
Puchner et al. (2021) [23]<br><br>Prospective cohort	N = 23 of which 14 underwent cognitive evaluations (mean age 57y; 70% M; 100% hospitalized)	- Logical Memory I & II of WMSIV<br>- VVM<br>- TAP	- HADS-D<br>- IES	- In 29% of the tested patients, cognitive deficits of concentration, memory, and/or executive functions were found.	- Lack of control group<br>- Small sample size
Soldati et al. (2021) [24]<br><br>Prospective cohort	N=23 patients (mean age: 53.6y; 78,2% M; 100% hospitalized in ICU)	- TICS-M (telephone interview)	- EuroQol (quality of life assessment)	- No patients with severe cognitive impairment<br>- 13% exhibited mild cognitive impairment<br>- Patients with mild cognitive impairment in TICS tended to have a low EuroQol score	- Lack of control group
Latronico et al. (2021) [25]<br><br>Prospective cohort	N = 114 patients (mean age 60y; 75% M; 100%	- MoCA	- SF-36<br>- HADS<br>- EMG	- N (%) of patients with Mild Cognitive Impairment= 23 (3 months); 16 (6 months); 7 (12 months)	- Single-centre study<br>- Follow-up evaluations

	hospitalized in ICU)			- N. (%) of patients with Moderate or Severe Cognitive Impairment = 2 (3 months); 1 (6 months); 0 (12 months)	possible in only half the sample
Venturelli et al. (2021) [26]<br><br>Prospective cohort	N = 767 (mean age: 63y; 67,1% M; 87% hospitalized with 8,6% of them requiring ICU admission)	- MoCA	- Full blood panel and clinical biochemistry<br>- IES-R<br>- HADS<br>- RSA	- MoCA was pathologic in just 2 out of the 304 patients who were tested	- Timeline of enrolment and assessments was not standardized<br>- Sample may not be representative of the entire affected population<br>- Lack of pre-COVID cognitive assessments
Mazza et al. (2021) [27]<br><br>Prospective cohort	N=226 (mean age 58.5y; 66% M; 78,3 % hospitalized)	- BACS (on 130 patients)	- Baseline systemic immune-inflammation index (SII)<br>- IES-R<br>- STAI-Y<br>- BDI-13<br>- ZSDS<br>- WHIIRS	- 16% were poor performers in at least one function, 17% in two, 14% in three, 11% in four, 5% in five, and 1.5% showed no good performance at all.<br>- Patients with psychopathology one-month after discharge performed worse on verbal fluency, information processing and executive functions at the three months assessment	- Cognitive assessment not performed in the entire sample<br>- Single-center study
Raman et al. (2021) [28]<br><br>Prospective cohort	N = 58 (mean age: 55.4y; 58,6% M; 100% hospitalized)<br><br>N of controls = 30 COVID -	- MoCA	- MRI scan<br>- GAD-7<br>- PHQ-9<br>- FSS<br>- Complete blood count and clinical biochemistry	- MoCA scores: ≤4 in 40% patients vs 16% in controls<br>- 28% had a total MoCA score that was abnormal compared to 17% of controls.<br>- The cognitive profile observed (primarily dysexecutive) among patients is also consistent with a vascular pattern, observed through MRI scans	- Small sample size<br>- Single-center study<br>- Lack of pre-COVID cognitive assessment and imaging<br>- Lack of follow-up
Dressing et al. (2021) [29]<br><br>Prospective cohort	N = 31 (mean age 53.6; 35,5% M; not hospitalized)	- MoCA	- Cerebral 18F-FDG PET	- The mean z scores of verbal and visual memory domains and composite z score were not significantly different from zero	- Small sample size<br>- Lack of pre-COVID cognitive assessments and imaging

				- The mean z scores for executive functions, attention and speed of processing were even higher than zero and, in total, almost 49% were completely unimpaired in the neurocognitive test battery<br>- MoCA performance= mild impairment was detected in 9 patients (29%; range, 23–25 point	
Van der Borst et al. (2021) [30]<br><br>Prospective cohort	N = 124 (mean age 59y; 60% M)	- TICS	- HADS<br>- CFQ<br>- PCL-5	- 15% of patients scored <34 on TICS	- Small sample size<br>- Single center study<br>- Lack of pre-COVID 19 cognitive assessments
Monti et al. (2021) [31]<br><br>Prospective cohort	N = 39 (mean age 56y; 90% M; 100% hospitalized)	- Itel-MMSE	- HADS<br>- EQ5D-3L<br>- PCL-5<br>- ISI	- After a median of 61 days after ICU discharge, only one patient (2.6%) had cognitive impairment at the Itel-MMSE scale	- Small sample size<br>- Short follow-up period<br>- Lack of face-to-face cognitive evaluations<br>- Single center study
Del Brutto et al. (2020) [32]<br><br>Prospective cohort	N = 93 (mean age 62.6y; 37% M)	- MoCA	- MRI<br>- EEG	- Cognitive decline in 21% of individuals with mild symptomatic SARS-CoV-2 infection, and only in 2% asymptomatic seronegative individuals.<br>- 13% individuals had a reduction in the post-pandemic MoCA that was ≥4 points larger than the reduction that occurred between two pre-pandemic MoCA assessments.<br>- Post-pandemic EEGs disclosed abnormalities in two individuals (both were SARS-CoV-2 seropositive and had cognitive decline).<br>- Post pandemic MRIs were normal in the 12 individuals with	- Small sample size<br>- Sample not stratified by age<br>- Use of cognitive screening tools and measures of general cognitive functioning<br>- Methodological bias (Scalp EEG recordings may miss an infrequent epileptiform activity or focal slowing)

				cognitive decline, including the two with abnormal EEGs	
Morin et al. (2021) [33]<br><br>Prospective cohort	N = 478 (mean age 60.9y; 57.9% M; 100% hospitalized)	- Q3PC cognitive screening questionnaire<br>- MoCA<br>- D2-R test	N/A	- Cognitive impairment was confirmed in 38.4% of patients, more commonly in patients aged 75 years or older<br>- Memory difficulties were reported by 17.5%, mental slowness by 10.1%, and concentration problems by 10% more than once a week	- Lack of control group<br>- Sample may not be representative of the entire population
Rass et al. (2021) [34]<br><br>Prospective cohort	N = 135 (mean age 56.0y; 61% M; 72,53% hospitalized)	- MoCA	- SF-36v2<br>- PCL5<br>- HADS	- Cognitive deficits were found in 23% of patients (in severe COVID-19 patients 29%, moderate 30%, mild 3%)<br>- 34% reported sleep disturbances 3 months after COVID-19.	- Lack of pre-COVID cognitive assessment<br>- Lack of long-term follow-up<br>- Sample not stratified by disease severity
Almeria et al. (2020) [35]<br><br>Prospective cohort	N = 35 (mean age 47.6y; 45,7% M)	- Global cognitive Index<br>- TAVEC<br>- WMS-IV<br>- TMT-A and B<br>- SDMT<br>- Stroop, Phonemic and Semantic fluency and Boston Naming Test from the NEURONORMA project	- Laboratory testing (D-dimer, ferritin)<br>- HADS	- Cognitive impairment in patients that required oxygen therapy during hospitalization<br>- Patients with headache and clinical hypoxia scored lower in the global Cognitive Index<br>- T- score lower than 30 was observed in memory domains, attention and semantic fluency and mental flexibility and in phonetic fluency	- Small sample size<br>- Evaluations performed right after the infection.
Cian et al. (2022) [36]<br><br>Prospective cohort	N = 29 COVID + (58,62% M, 100% hospitalized)<br><br>N of controls COVID - = 29	-MMSE<br>-RAVLT<br>-CPM47<br>-CDT<br>-The phonemic/semantic and alternate fluency test<br>-Digit Span Forward and Backward	- STAI<br>- BDI-II	- Significant differences were found between groups in the RAVLT scores (learning, recall, and recognition)<br>- Significant difference between groups in Digit backward test<br>- The number of people with at least one pathological score was higher in the COVID+ group than in controls	- Small sample size.<br>- Methodological bias (exploratory study).<br>- Lack of face-to-face cognitive evaluations<br>- Lack of neuroimaging correlates of the findings
De Lorenzo et al. (2020) [37]<br><br>Prospective and retrospective cohort study	N = 185 (mean age 57y; 66,5% M; 68,1% hospitalized)	- MoCA	- WHOQOL<br>- IER<br>- STAI<br>- WHIIRS	At follow-up, 25.4% achieved MoCA scores compatible with cognitive impairment	N/A

Walle-Hansen et al. (2021) [38]<br><br>Retrospective cohort study	N = 106 patients (mean age 74,3y; 57% M; 100% hospitalized)	-MoCA	- EQ-5D-5L<br>- ADL<br>- SPPB	- The mean sum scores of both MoCA and SPPB were lower in the oldest age group<br>- 43% of the patients experienced a negative change in cognitive function 6 months after the COVID-19 hospitalization (more cognitive decline among persons >75y compared to younger persons)	- Short follow-up period; single follow-up evaluation<br>- Recall bias for the pre-COVID assessment<br>- Sample with same disease severity
Patel et al. (2021) [39]<br><br>Retrospective cohort study	N = 77 (mean age 61.03y; 63,6%M; 31,8% acute hospitalization)	- MoCA	- QI-SC	- 80.5% demonstrated cognitive deficits on the MoCA at admission: 51% mild deficits, 26% moderate deficits and 4% severe deficits.<br>- At discharge, 78% continued to exhibit cognitive impairment on the MoCA.<br>- The 45 patients with admission and discharge MoCA scores improved on the MoCA	- Lack of discharge cognitive data<br>- Sample with same disease severity
Manera et al. (2021) [40]<br><br>Retrospective cohort study	N = 152 (mean age 67.0y; 66,4% M; 48,7% ICU)	- MMSE	- NA	- Impaired MMSE performances were highly prevalent in mild-to-moderate patients (26.3%)<br>- Below-cutoff MMSE percentage was visibly higher in Neuro+ (16.5%) vs. Neuro− (4.1%) patients.<br>- Within severity degrees, impaired MMSE performances were notably more frequent for mild-to-moderate (26.3%).<br>- A trend toward a lower prevalence of defective MMSE scores was detected in ICU-admitted patients (19.2%)—when descriptively compared to those not admitted (5.4%).<br>- ICU admission predicted a higher probability of responding correctly to constructional praxis	- Use of screening tools and measures of general cognitive functioning

				item—when compared to non-admission	
Sardella et al. (2022) [41]<br><br>Cross-sectional study	N = 71 (mean age 80,7 y; 30% M; not hospitalized)	- Itel-MMSE (Italian telephone version)	- BADL<br>- IADL<br>- SF-12<br>- PCS<br>- MCS	- Patients reported significantly lower scores on the MMSE at t2 compared to the scores obtained at baseline	- Small sample size<br>- Sample not stratified by age<br>- Use of cognitive screening tools and measures of general cognitive functioning
Abdelghani et al. (2022) [42]<br><br>Cross-sectional study	N = 85 patients (mean age: 35.95y; 18.8 % M; not hospitalized-asymptomatic)<br><br>N of controls = 85 (mean age: 33.68y; 27.1 % M)	- MoCA	- HADS	- Patients were more likely to have cognitive impairment than the control subjects<br>- Patients had a significant decline in visuo-executive skills, naming, attention, language, abstraction, and delayed recall<br>- Even after being adjusted for associated anxiety and depressive symptoms, patients had greater odds of cognitive impairment	- Methodological bias (cross-sectional design)<br>- Single-center design<br>- Small sample size<br>- Use of cognitive screening tools and measures of general cognitive functioning
Aiello et al. (2022) [43]<br><br>Cross-sectional study	N = 54 of which 37 RCD* + (mean age 70.30y; 40,5% M) and 17 RCD* – (mean age 69.59y; 58,8% M)<br><br>*at least one neurological/ psychiatric condition possibly affecting cognition	- MMSE<br>- ACE-R<br>- FAB<br>- Attentional Matrices (28 patients)	- N/A	- Prevalence of defective MMSE scores was<br>- 24.3% in RCD + patients and 5.9% in the RCD – group.<br>- ACE-R-total below-cutoff scores were less frequent (RCD + 5.4%; RCD – 5.9%).<br>- In both groups, no effects of disease severity, ICU admission, steroidal treatment, and co-occurring infection were detected on adjusted cognitive scores—except for cooccurring infections on ACE-R-F and ICU admission rates on FAB-3 scores in RCD – patients	- Small sample size<br>- Use of cognitive screening tools and measures of general cognitive functioning
Bolattürk et al. (2022) [44]<br><br>Cross-sectional study	N = 40 patients (mean age: 51.3y; 55 % M; 100% hospitalized)	- MoCA<br>- MMSE	- PSQI<br>- HAM-A<br>- HAM-D	- Early-stage cognitive impairment was detected in 15% of patients<br>- MMSE was normal in 85% of patients and the mean MMSE score of	- Small sample size<br>- Short follow-up period<br>- Lack of control group

				the patients was 26.9±2.1<br>- MoCA test was positive in 55% of the patients, and the mean MoCA score of the patients was 19.6±5.2<br>- Significant correlation of MoCA scores and HAM-D	
Cecchetti et al. (2022) [45]<br><br>Cross-sectional study	N = 49 (baseline) and 33 (follow-up) (mean age 60.8y; 73,4% M; 85,7% hospitalized)<br><br>N of controls = 36 (for cognitive and MRI); 33 (for EEG)	- MMSE<br>- FAB<br>- SDMT<br>- TMT-A and B<br>- RAVLT<br>- Digit span forward and backward<br>- VOSP<br>- SAND	- EEG<br>- MRI (3T) (36 patients)	- 53% of patients had disturbances in at least one cognitive domain 2 months after COVID-19 resolution with a main involvement of the executive functions<br>- The most affected domains were executive functions, memory and visual-spatial, domain<br>- 25% of subjects showed a multidomain impairment<br>- At follow-up, 36% of patients showed an impairment in at least one cognitive domain<br>- Compared with healthy controls, patients performed worse in all investigated domains	- Small sample size<br>- Lack of pre-COVID cognitive, EEG or MRI assessments<br>- - Methodological bias (19-channel EEG has a low spatial resolution and precluded the CSD and LLC analyses at sub-regional level; longitudinal MRI data was not acquired)<br>- A control cohort with subacute respiratory dysfunction or viral infections different from COVID-19 was not enrolled
Guo et al. (2022) [46]<br><br>Cross-sectional study	N = 421(181 patients COVID +; 28,2% M)<br><br>N of controls = 185 patients (36,2% M)	- WCST<br>- Pictorial Associative Memory Test<br>- Category Fluency Test<br>- Word List Recognition Memory Test<br>- 2D Mental Rotation Test<br>- Number Counting Test<br>- Relational Reasoning test	- NA	- There was a significant negative influence of the COVID-19 infection on memory performance, even when controlling for age, sex, country, and education level.	- Methodological bias (exploratory study)<br>- Lack of face-to-face cognitive evaluation<br>- Sample not stratified by disease severity

Henneghan et al. (2022) [47]<br><br>Cross- sectional study	N = 72 patients (mean age 36y; 26% M; not hospitalized)	- BrainCheck (web-based battery):<br>o TMT<br>o Digit Symbol Substitution Test<br>o Stroop Test<br>o List Learning Test<br>- PROMIS Cognitive (subjective)	- PROMIS 57<br>- Perceived Stress Scale	- Results indicated that 40% of participants demonstrated objective cognitive impairment. The largest number of participants showed impairment on executive functions<br>- Median percentage of people with cognitive impairment = 61%<br>- Incidence of cognitive impairment is lower in mild-to-moderate cases<br>- Executive function was the most affected cognitive domain<br>- Greater frequency of impairment on a test of attention and processing speed in males<br>- Moderate severity disease was correlated with attention/processing speed impairment<br>- Younger age was correlated with objective cognitive impairment and higher perceived stress, anxiety and depressive symptoms	- Methodological bias (cross-sectional study)<br>- Lack of control group<br>- Recall bias (lack of pre-COVID cognitive assessment)<br>- Lack of face-to-face cognitive evaluation<br>- Sample may not be representative of the entire affected population
Serrano- Castro et al. (2022) [48]<br><br>Cross-sectional study	N = 152 cases (mean age 71y: 37% M)<br><br>N of controls = 40 (mean age 52.2y; 50% M)	- MoCA<br>- TAVEC<br>- FCRST<br>- BNT<br>- DRT<br>- TMT A and B<br>- FAS<br>- RCFT	- Complete blood count and biochemistry<br>- Proinflammatory chemokines and growth factors<br>- STAI<br>- BDI-II	- Impairment in episodic verbal memory was observed in 34.7% to 38.5%<br>- Working memory was affected in 26.4–36.7% of the sample<br>- The scores obtained for attention and orientation were abnormal	- Absence of neuroradiological data
Becker et al. (2021) [49]<br><br>Cross-sectional study	N = 740 (mean age 49.0y; 37% M; 27% hospitalized)	- Number Span forward and backward<br>- TMT-A and B<br>- Phonemic and category fluency<br>- Hopkins Verbal Learning Test–Revised	- NA	Impaired total:<br>- 10% attention<br>- 10% working memory<br>- 18% processing speed<br>- 16% executive functions<br>- 15% phonemic fluency<br>- 20% category fluency<br>- 24% memory encoding<br>- 23% memory recall<br>- 10% memory recognition<br><br>-Hospitalized patients were more likely to have impairments in attention,	- Potential sampling bias

				executive functions, category fluency, memory encoding and memory recall than those in the outpatient group.	
Ermis et al. (2021) [50]<br><br>Cross-sectional study	N=53 (mean age 63y; 60% M; 100% hospitalized)	- MoCA (in acute phase)	- CSF exam<br>- Brain CT or MRI<br>- EEG	- Most of the tested patients (61.5%) showed cognitive impairment with deficits primarily in executive function, attention, language and delayed recall	- Evaluations performed right after the infection.
Jaywant et al. (2021) [51]<br><br>Cross-sectional study	N = 57 (mean age 64.5y; 75% M; 100% hospitalized)	- BMET	N/A	- 81% had cognitive impairment:<br>○ 55% in working memory<br>○ 47% in set-shifting<br>○ 46% in divided attention<br>○ 40% in processing speed	- Single-center study<br>- Sample may not be representative of the entire affected population<br>- Lack of control group<br>- Not all patients completed all subtests of the BMET
Albu et al. (2021) [52]<br><br>Cross-sectional study	N = 30 (mean age 54y; 63,3% M; 100% hospitalized)	- Barcelona Test Digit Span forward and backward<br>- RAVLT<br>- PMR task	- HADS	- Cognitive impairment was found in 63.3% of patients, with a similar profile in both subgroups.	- Sample may not be representative of the entire affected population<br>- Lack of pre-COVID 19 cognitive assessments
Ferrucci et al. (2021) [53]<br><br>Cross-sectional study	N = 38 (mean age 53,45y; 71% M; 100% hospitalized)	- MoCA<br>- BRB-NT:<br>○ SRT<br>○ SPART<br>○ SDMT<br>○ PASAT<br>○ WLG	- BDI-II<br>- SSD questionnaire	- 42.1% showed processing speed deficits<br>- 26.3% showed delayed verbal recall deficits<br>- 10.5% showed deficits in immediate verbal recall<br>- 18.4% showed deficits in visual long-term memory<br>- 15.8% showed deficits in visual short-term memory<br>- 7.9% showed deficits in semantic verbal fluency	- Lack of control group<br>- Lack of pre-COVID cognitive assessment<br>- Sample not stratified by gender
Johnsen et al. (2021) [54]	N = 57 patients (mean age 51 y; 28% M; 44% hospitalized,	- SCIP-D<br>- TMT-B	- WPAI<br>- EQ-5D-5L<br>- CFQ	- The percentage of patients with clinically significant cognitive	- Small sample size<br>- Potential selection bias

Cross-sectional study	36% not hospitalized)<br><br>*N of patients who received cognitive evaluation= 45			impairment ranged from 51% to 58%<br>- 38–53% of patients showed broad impairments<br>- 4–16% patients showed selective impairments;	- Clinical and laboratory data from non-hospitalized patients during the acute phase were not available.
Liu et al. (2021) [55]<br><br>Cross-sectional study	N = 1539 (mean age 69y; 47,95% M)<br>N of controls = 466 (mean age 67y; 48,5%M)	- TICS-40 (telephone)<br>- IQCODE (subjective, family questionnaire)	- NA	- Compared with controls, COVID-19 patients had lower TICS-40 scores and higher IQCODE scores<br>- Severe COVID-19 patients had lower TICS-40 scores and higher IQCODE scores than non-severe COVID-19 patients<br>- Severe COVID-19 patients had a higher proportion of cases with current cognitive impairment and longitudinal cognitive decline than non-severe COVID-19 patients<br>- The severity of COVID-19 and ICU admission were found to be associated with an increased risk of cognitive impairment.	- Lack of face-to-face cognitive assessment<br>- Lack of pre-COVID cognitive assessment<br>- A control cohort with subacute respiratory dysfunction or viral infections different from COVID-19 was not enrolled<br>- Potential selection bias<br>- Control group is not matched to sample group
Hellgren et al. (2021) [56]<br><br>Cross-sectional study	N = 35 (median age 59y; 80% M; 100% hospitalized)	- RBANS	- HADS<br>- MFI<br>- Brain MRI	- 46% showed cognitive impairments<br>o 17% showed mildly/moderately impaired cognition<br>o 29% had severely impaired cognition<br>- Immediate Memory and Delayed Memory were the indices where most patients performed below cut-off	- Lack of global cognitive assessment<br>- Lack of pre-COVID cognitive assessment<br>- Small sample size<br>- Lack of control group
Méndez et al. (2021) [57]<br><br>Cross-sectional study	N = 179 (mean age 57y; 58,7% M; 100% hospitalized)	- Delayed memory subtests from SCIP<br>- ANT from COWAT<br>- Digit Span backward from WAIS-III	- GAD-7<br>- PHQ-2<br>- DTS<br>- SF-12	- 38% of patients presented moderate impairment and 11.2% severe impairment in immediate verbal memory<br>- 11.8% of survivors had moderate impairment and 2.8% had severe impairment in delayed verbal memory	- Single-center study<br>- Lack of face-to-face cognitive evaluations<br>- Large number of dropouts

				- Working memory was moderately impaired in 6.1% and severely impaired in 1.1% of survivors<br>- 58.7% patients met criteria for moderate neurocognitive impairment and 18.4% for severe neurocognitive impairment	
Versace et al. (2021) [58]<br><br>Cross-sectional study	N= 12 (mean age 67y; 83,3% M; 100% hospitalized)	- FAB	- FRS	- Diminished executive functions, as documented by abnormal scores corrected for age and education on the FAB	- Small sample size with sequelae of inhomogeneous neurological affections
Woo et al. (2020) [59]<br><br>Cross-sectional study	N = 18 (mean age 42.2y; 44,4% M; 61% hospitalized)<br><br>N of controls = 18 (mean age 45.8y)	- TICS-M (telephone)	- PHQ-9<br>- FAS<br>- Analysis of serological parameters during acute COVID-19<br>- Analysis of cerebrospina l fluid (CSF)<br>- Cranial imaging	- Post-COVID-19 patients scored significantly lower results in the TICS-M compared to healthy controls<br>- 50% reported attention deficits<br>- 44.4% reported concentration deficits<br>- 44.4% reported short-term memory deficits<br>- 27.8% reported troubles in finding words	- Small sample size.<br>- Confounders for cognitive testing such as years of education and substance abuse were not assessed.
Zhou et al. (2020) [60]<br><br>Cross-sectional study	N =29 (mean age 47.0y; 62% M)<br><br>N of controls = 29 healthy volunteers (matched by age, gender, education)	- TMT<br>- SCT<br>- CPT (Part 1,2,3)<br>- Digit span	- GAD-7<br>- PHQ-9<br>- Blood tests (IL-2/ IL-4/ IL-6/ IL10/ TNF-α/ IFN-γ; CRP)	- The COVID-19 patients had a lower correct number CPT 2 and CPT 3 compared with the controls<br>- There was no significant difference between the two groups in TMT, SCT, or Digit span	- Small sample size<br>- Potential selection bias due to inclusion criteria
Hadad et al. (2022) [61]<br><br>Cross-sectional study	N= 46 (mean age 50y; 35% M; 67% not hospitalized)	- MoCA	- Brain CT scan<br>- MRI<br>- EEG (in 5 patients)<br>- ADL<br>- IADL	- The total MoCA score of the patients was not statistically different from controls<br>- There was a statistically not significant correlation between the MoCA index scores and disease severity, except a trend-level association with the memory index (<br>- Executive function, language and attention index scores were	- Single-center study<br>- Not all patients attended follow-up evaluation<br>- Lack of a control group<br>- Heterogeneous sample (sample not stratified by age, background and disease severity)

				significantly worse compared to the older normative sample<br>- EEG/ MRI/ CT evaluations were normal in all patients tested	- Normative cognitive data resulted from a group with different social and ethno-racial background<br>- Not all the individuals received the same tests
Miskowiak et al. (2022) [62]<br><br>Cross sectional study	N at baseline = 71<br><br>N at three months follow up= 29 (included for cognition assessment)<br><br>N at one year follow up= 25 included for cognition assessment<br><br>(mean age 56y: 52% M; 100% hospitalized)	- SCIP-D<br>- TMT-B	- WPAI<br>- EQ-5D-5L<br>- ED5D<br>- HDRS-17<br>- CFQ	- 56% reported cognitive impairments compared with their expected:<br>○ 48 % fulfilled the criterion for global impairment<br>○ 8% were selectively impaired<br>○ 44% were cognitively normal<br>- In comparison with HC sample, 48% were identified as cognitively impaired:<br>○ 40% with global impairment<br>○ 8% with selective impairment<br>○ 52% were cognitively normal.<br>- Large effect size on the working memory test<br>- Moderate to large impairments in verbal learning test - immediate, verbal fluency test and psychomotor speed test.	- Small sample size<br>- Lack of control group<br>- Use of screening tools and measures of general cognitive functions
Amalakanti et al. (2021) [63]<br><br>Case-control study	N = 93 (mean age 36.2y; 47,7% M; not hospitalized)<br><br>N of controls = 102 (mean age 35.6y; 45,3% M)	- MoCA	N/A	- There was no significant difference in the overall cognitive assessment scores between the two groups<br>- COVID-19 patients secured lower scores than controls in the domains of visuoperception, naming and fluency<br>- COVID positive subjects aged greater than 50 years scored lower in the MoCA when compared to the younger people	- Small sample size

Ortelli et al. (2021) [64]<br><br>Case-control study	N =12 patients (mean age 67y; 83% M; 100% hospitalized)<br><br>N of controls = 12, matched by age and sex	- MoCA<br>- FAB<br>- Vigilance Task<br>- Stroop Interference Task<br>- Navon Task	- CRP and IL-6 serum levels<br>- FRS and FSS (fatigue assessment)<br>- BDI<br>- Apathy Evaluation Scale<br>- TMS (central motor excitability assessment)	- MoCA: 15.5/30 (mean score in post-COVID19 patients)<br>- The most affected was the executive domain<br>- FAB: 13.4/18 (mean score in post COVID19 patients), demonstrating evidence of a dysexecutive syndrome	- Lack of long-term follow-up period
Tolentino et al. (2021) [65]<br><br>Case report	N = 1 (age 47y; M; hospitalized)	- MMSE<br>- CVAT	- GAD-7<br>- PHQ-9	- The patient suffered from a more limited dysfunction involving the attentional system<br>- A worsening in attention performance on Day 6 preceded the maximum drop in the patient's oxygen saturation	- N/A
Yesilkaya et al. (2021) [66]<br><br>Case report	N = 1 (age 29y; M; hospitalized)	- FAB<br>- TMT-A and B<br>- CVLT	- GDS<br>- EEG<br>- MRI<br>- CT	- Impairment in memory, executive functioning, motor programming, attention, and concentration<br>- No abnormalities on EEG, conventional MRIs and CT.<br>- No neurologic nor cognitive deficits were detected at the patient's three months follow-ups.	- N/A
Hellmuth et al. (2021) [67]<br><br>Case series	N = 2 (mean age 44.5y; 100% F)	- MoCA<br>- CVLT<br>- MMSE<br>- Digit span forward and backwards<br>- D-KEFS<br>- TMT<br>- RCFT<br>- NAB	N/A	- RCFT<br>o Cases: 33/36 low average<br>- Figure 2 min delay<br>o Cases: 16/36 below average<br>- Backward Span<br>o Cases: 4 low average<br>- Inhibition/switching<br>o Cases: 77 low average	- N/A
Whiteside et al. (2021) [68]<br><br>Case series	N = 3 (mean age 70y; 66,7% M; 100% hospitalized)	- Vocabulary Subtest (WAIS-IV)<br>- RDS<br>- HVLT-R<br>- RBANS<br>- Complex Ideational Material subtest from BDAE	- ILS<br>- BAI<br>- GDS	- Neurocognitive deficits particularly in encoding and verbal fluency	- Small sample size<br>- Lack of face-to-face cognitive evaluations

		- O-TMT<br>- TSAT			
Gautam et al. (2021) [69]<br><br>Case series	N = 200 (mean age 56.5y; 62,5% M; 100% hospitalized)	- MoCA	- EQ-5D-5L	- In 12.5% of patients, some cognitive impairment was noted, mainly in concentration and short-term recall	- Small sample size<br>- Single center study<br>- Sample with same disease severity
Beaud et al. (2020) [70]<br><br>Case series	N = 13 (mean age 64,7y; 77% M; 100% hospitalized)	- MoCA<br>- FAB	- MRI	- MoCA revealed mild (4 subjects) and moderate-severe (5 subjects) deficits<br>- The most affected were executive, memory, attentional and visuospatial functions<br>- FAB revealed executive dysfunction in eight patients<br>- The most affected subtest was lexical fluency<br>- Cognitive impairment in severe COVID-19, does not correlate with length of mechanical ventilation or length of ICU stay and thus severity of the acute illness.	- N/A
Groiss et al. (2020) [71]<br><br>Case series	N = 4 (age 59.5y; 100% M; 100% hospitalized)	- MoCA<br>- SDMT<br>- MMSE	- EEG	- All patients showed clinically relevant impairment of cognition<br>- All patients showed signs of central nervous system affection	- Small sample size<br>- Use of cognitive screening tools and measures of general cognitive functioning<br>- Lack of pre-COVID cognitive assessments
Negrini et al. (2020) [72]<br><br>Case series	N = 9 (mean age 60y; 67% M; 100% hospitalized)	- MMSE<br>- FAB	- STAI<br>- BDI	- 33.3% had a pathologic MMSE score<br>- Lower scores were registered in the domain of attention and calculation, short-term memory, constructional praxia and written language<br>- The cognitive decay appeared to be linearly	- Lack of long-term follow-up period<br>- Small sample size

				associated with the length of stay in the ICU	

ACE-R= Addenbrooke's Cognitive Examination—Revised; **ADL=** Activities of daily living; **ANT=** Animal Name Testing; **BACS=** Brief Assessment of Cognition in Schizophrenia; **BADL=** Bristol Activities of daily living; **BAI=** Beck Anxiety Inventory; **BDAE=** Boston Diagnostic Aphasia Examination; **BDI**= Beck Depression Inventory; **BMET=** Brief Memory and Executive Test; **BNP=** Brain Natriuretic Peptide; **BNT**= Boston Naming Test; **BRB-NT**=Brief Repeatable Battery of Neuropsychological Tests; **BVMT-R=** Brief Visuospatial Memory Test-Revised**; CDT=** Clock Drawing Test; **CFQ=** Cognitive Failures Questionnaire; **CFS=** Clinical Frailty Scale; **CK=** Creatine Kinase**; COWA=** Controlled Oral Word Association by categories; **CPM47=** Colored Progressive Matrices 47; **CPT=** Continuous Performance Test; **CRP=** C-Reactive Protein; **CVLT=** California Verbal Learning Test; **CVAT=** Continuous Visual Attention Test; **D-KEFS=** Delis-Kaplan Executive Functions Test; **DASS-21**= Depression, Anxiety and Stress Scale 21 items; **DRT=** Digit Retention Test; **DTS=** 17- Items Davidson Trauma Scale; **EEG**= Electroencephalography; **EQ-5D-5L** = 5-level EuroQol-5 Dimension; **FAB=** Frontal Assessment Battery; **FAS=** Fatigue Assessment Scale; **FAS=** Verbal Fluency Test; **FCRST=** Free and Cued Selective Reminding Test; **FDG-PET=** Fluorodeoxyglucose Positron Emission Tomography; **FIC=** Functional Impairment Checklist; **FRS=** Fatigue Rating Scale; **FSS=** Fatigue Severity Scale; **FWIT=** Color-Word Interference Test; **GAD-7** = Generalized Anxiety Disorder-7; **GDS=** Global Deterioration Scale; **GDS=** Geriatric Depression Scale 15-item version; **HADS=** Hospital Anxiety and Depression Scale; **HDRS-17=** Hamilton Depression Rating scale 17-items; **HVLT=** Hopkins Verbal Learning Test-Revised; **IADL=** Lawton-Brody Instrumental Activities of Daily Living; **ICU**= Intensive Care Unit; **IES-6** = Impact of Events Scale-6; **IES-R=** Impact of Events Scale – Revised; **ILS=** Independent Living Scales; **IQCODE=** Informant Questionnaire on Cognitive Decline in the Elderly; **LDH=** Lactate De-hydrogenase; **MADRS=** Montgomery and Asberg Depression Scale; **MCS=** Mental Component Summary**; MFI=** Multi-dimensional Fatigue Inventory; **MMSE=** Mini Mental State Exam; **MoCA=** Montreal Cognitive Assessment; **MRI=** Magnetic Resonance Imaging; **N/A=** Not Available; **NAB=** Neuropsychological Assessment Battery; **NIH-Toolbox=** National Institutes of Health Toolbox; **NPI=** Neuropsychiatry Inventory; **OMC=** Orientation-Memory-Concentration Test; **OCD=** obsessive-compulsive disorder according to DSM-V; **PASAT=** Paced Auditory Serial Addition Test**; PCL5**= Posttraumatic Stress Disorder Checklist–5; **PCS=** Physical Component Summary; **PHQ-9** = Patient Health Questionnaire-9; **PROMIS=** Patient-Reported Outcomes Measurement Information System; **PSQI=** Pittsburgh Sleep Quality Index; **QDRS** = Quick Dementia Rating Scale; **QI-SC=** Quality Indicator For Self-Care; **RAVLT** = Rey Auditory Verbal Learning Test; **RBANS=** Repeatable Battery for the Assessment of Neuropsychological Status; **RCFT**= Rey Complex Figure Test; **RDS=** Reliable Digit Span; **RSA=** Resilience Scale for Adults; **SAND**= Screening for aphasia in neurodegeneration; **SCIP-D=** Screen for Cognitive Impairment in Psychiatry Danish Version; **SCT=** Sign Coding Test; **SDMT**= Symbol Digit Modalities Test; **SDSSS=** Stress Disorder Symptom Severity Scale according to the DSM-V; **SF-12=** Short-Form Health Survey 12 item; **SF-36=**36-Item Short-Form Health Survey; **SPART=** 10/36 Spatial Recall Test; **SPHERE-34=** Somatic and Psychologic Health Report-34 item; **SPPB=** Short Physical Performance Battery; **SRT**= Selective Reminding Test; **STAI=** State - Trait Anxiety Inventory; **TAP=** Test of Attentional Performance; **TAVEC**= Test de Aprendizaje Verbal Espana-Complutense; **TICS-40=** Telephone Interview of Cognitive Status-40; **TICS-M=** Telephone Interview of Cognitive Status; **TMT-A**= Trail Making Test-A; **TMT-B**= Trail Making Test-B; **TMT=** Trail Making Test; **TSAT=** Test of Sustained Attention and Tracking; **VVM** =Verbal and visual memory test; **VOSP**= Visual object and space perception battery; **WCST=** Wisconsin Cart Sorting Test; **WHIIRS**= Women's Health Initiative Insomnia Rating Scale; **WLG=** Word List Generation Test; **WMS-IV**= Visual Reproduction of the Wechsler Memory Scale –IV; **WPAI=** Work Productivity and Activity Impairment Questionnaire; **ZSDS=** Zung Self-Rating Depression Scale.

- **References**

1. Ceban, F.; Ling, S.; Lui, L.M.W.; Lee, Y.; Gill, H.; Teopiz, K.M.; Rodrigues, N.B.; Subramaniapillai, M.; Di Vincenzo, J.D.; Cao, B.; et al. Fatigue and cognitive impairment in Post-COVID-19 Syndrome: A systematic review and meta-analysis. *Brain, Behavior, and Immunity* **2022**, *101*, 93-135, doi:https://doi.org/10.1016/j.bbi.2021.12.020.
2. Crivelli, L.; Palmer, K.; Calandri, I.; Guekht, A.; Beghi, E.; Carroll, W.; Frontera, J.; García-Azorín, D.; Westenberg, E.; Winkler, A.S.; et al. Changes in cognitive functioning after COVID-19: A systematic review and meta-analysis. *Alzheimers Dement* **2022**, *18*, 1047-1066, doi:10.1002/alz.12644.
3. Tavares-Júnior, J.W.L.; de Souza, A.C.C.; Borges, J.W.P.; Oliveira, D.N.; Siqueira-Neto, J.I.; Sobreira-Neto, M.A.; Braga-Neto, P. COVID-19 associated cognitive impairment: A systematic review. *Cortex* **2022**, *152*, 77-97, doi:https://doi.org/10.1016/j.cortex.2022.04.006.
4. Schou, T.M.; Joca, S.; Wegener, G.; Bay-Richter, C. Psychiatric and neuropsychiatric sequelae of COVID-19 – A systematic review. *Brain, Behavior, and Immunity* **2021**, *97*, 328-348, doi:https://doi.org/10.1016/j.bbi.2021.07.018.
5. Vanderlind, W.M.; Rabinovitz, B.B.; Miao, I.Y.; Oberlin, L.E.; Bueno-Castellano, C.; Fridman, C.; Jaywant, A.; Kanellopoulos, D. A systematic review of neuropsychological and psychiatric sequalae of COVID-19: implications for treatment. *Curr Opin Psychiatry* **2021**, *34*, 420-433, doi:10.1097/yco.0000000000000713.
6. Altuna, M.; Sánchez-Saudinós, M.B.; Lleó, A. Cognitive symptoms after COVID-19. *Neurology Perspectives* **2021**, *1*, S16-S24, doi:https://doi.org/10.1016/j.neurop.2021.10.005.
7. Daroische, R.; Hemminghyth, M.S.; Eilertsen, T.H.; Breitve, M.H.; Chwiszczuk, L.J. Cognitive Impairment After COVID-19-A Review on Objective Test Data. *Front Neurol* **2021**, *12*, 699582, doi:10.3389/fneur.2021.699582.
8. Rabinovitz, B.; Jaywant, A.; Fridman, C.B. Neuropsychological functioning in severe acute respiratory disorders caused by the coronavirus: Implications for the current COVID-19 pandemic. *The Clinical Neuropsychologist* **2020**, *34*, 1453-1479, doi:10.1080/13854046.2020.1803408.
9. Weihe, S.; Mortensen, C.B.; Haase, N.; Andersen, L.P.K.; Mohr, T.; Siegel, H.; Ibsen, M.; Jørgensen, V.R.L.; Buck, D.L.; Pedersen, H.B.S.; et al. Long-term cognitive and functional status in Danish ICU patients with COVID-19. *Acta Anaesthesiologica Scandinavica* **2022**, *66*, 978-986, doi:https://doi.org/10.1111/aas.14108.
10. Bonizzato, S.; Ghiggia, A.; Ferraro, F.; Galante, E. Cognitive, behavioral, and psychological manifestations of COVID-19 in post-acute rehabilitation setting: preliminary data of an observational study. *Neurological Sciences* **2022**, *43*, 51-58, doi:10.1007/s10072-021-05653-w.
11. Holdsworth, D.A.; Chamley, R.; Barker-Davies, R.; O'Sullivan, O.; Ladlow, P.; Mitchell, J.L.; Dewson, D.; Mills, D.; May, S.L.J.; Cranley, M.; et al. Comprehensive clinical assessment identifies specific neurocognitive deficits in working-age patients with long-COVID. *PLOS ONE* **2022**, *17*, e0267392, doi:10.1371/journal.pone.0267392.
12. Rubega, M.; Ciringione, L.; Bertuccelli, M.; Paramento, M.; Sparacino, G.; Vianello, A.; Masiero, S.; Vallesi, A.; Formaggio, E.; Del Felice, A. High-density EEG sleep correlates of cognitive and affective impairment at 12-month follow-up after COVID-19. *Clinical Neurophysiology* **2022**, *140*, 126-135, doi:https://doi.org/10.1016/j.clinph.2022.05.017.
13. Vialatte de Pémille, C.; Ray, A.; Michel, A.; Stefano, F.; Yim, T.; Bruel, C.; Zuber, M. Prevalence and prospective evaluation of cognitive dysfunctions after SARS due to SARS-CoV-2 virus. The COgnitiVID study. *Revue Neurologique* **2022**, *178*, 802-807, doi:https://doi.org/10.1016/j.neurol.2022.03.014.
14. García-Sánchez, C.; Calabria, M.; Grunden, N.; Pons, C.; Arroyo, J.A.; Gómez-Anson, B.; Lleó, A.; Alcolea, D.; Belvís, R.; Morollón, N.; et al. Neuropsychological deficits in patients with cognitive complaints after COVID-19. *Brain and Behavior* **2022**, *12*, e2508, doi:https://doi.org/10.1002/brb3.2508.

15. Vannorsdall, T.D.; Brigham, E.; Fawzy, A.; Raju, S.; Gorgone, A.; Pletnikova, A.; Lyketsos, C.G.; Parker, A.M.; Oh, E.S. Cognitive Dysfunction, Psychiatric Distress, and Functional Decline After COVID-19. *J Acad Consult Liaison Psychiatry* **2022**, *63*, 133-143, doi:10.1016/j.jaclp.2021.10.006.
16. Frontera, J.A.; Yang, D.; Lewis, A.; Patel, P.; Medicherla, C.; Arena, V.; Fang, T.; Andino, A.; Snyder, T.; Madhavan, M.; et al. A prospective study of long-term outcomes among hospitalized COVID-19 patients with and without neurological complications. *Journal of the Neurological Sciences* **2021**, *426*, 117486, doi:https://doi.org/10.1016/j.jns.2021.117486.
17. Evans, R.A.; McAuley, H.; Harrison, E.M.; Shikotra, A.; Singapuri, A.; Sereno, M.; Elneima, O.; Docherty, A.B.; Lone, N.I.; Leavy, O.C.; et al. Physical, cognitive, and mental health impacts of COVID-19 after hospitalisation (PHOSP-COVID): a UK multicentre, prospective cohort study. *The Lancet Respiratory Medicine* **2021**, *9*, 1275-1287, doi:10.1016/S2213-2600(21)00383-0.
18. Miskowiak, K.W.; Johnsen, S.; Sattler, S.M.; Nielsen, S.; Kunalan, K.; Rungby, J.; Lapperre, T.; Porsberg, C.M. Cognitive impairments four months after COVID-19 hospital discharge: Pattern, severity and association with illness variables. *European Neuropsychopharmacology* **2021**, *46*, 39-48, doi:https://doi.org/10.1016/j.euroneuro.2021.03.019.
19. Graham, E.L.; Clark, J.R.; Orban, Z.S.; Lim, P.H.; Szymanski, A.L.; Taylor, C.; DiBiase, R.M.; Jia, D.T.; Balabanov, R.; Ho, S.U.; et al. Persistent neurologic symptoms and cognitive dysfunction in non-hospitalized Covid-19 “long haulers”. *Annals of Clinical and Translational Neurology* **2021**, *8*, 1073-1085, doi:https://doi.org/10.1002/acn3.51350.
20. Mattioli, F.; Stampatori, C.; Righetti, F.; Sala, E.; Tomasi, C.; De Palma, G. Neurological and cognitive sequelae of Covid-19: a four month follow-up. *Journal of Neurology* **2021**, *268*, 4422-4428, doi:10.1007/s00415-021-10579-6.
21. Hosp, J.A.; Dressing, A.; Blazhenets, G.; Bormann, T.; Rau, A.; Schwabenland, M.; Thurow, J.; Wagner, D.; Waller, C.; Niesen, W.D.; et al. Cognitive impairment and altered cerebral glucose metabolism in the subacute stage of COVID-19. *Brain* **2021**, *144*, 1263-1276, doi:10.1093/brain/awab009.
22. Leth, S.; Gunst, J.D.; Mathiasen, V.; Hansen, K.; Søgaard, O.; Østergaard, L.; Jensen-Fangel, S.; Storgaard, M.; Agergaard, J. Persistent Symptoms in Patients Recovering From COVID-19 in Denmark. *Open Forum Infect Dis* **2021**, *8*, ofab042, doi:10.1093/ofid/ofab042.
23. Puchner, B.; Sahanic, S.; Kirchmair, R.; Pizzini, A.; Sonnweber, B.; Wöll, E.; Mühlbacher, A.; Garimorth, K.; Dareb, B.; Ehling, R.; et al. Beneficial effects of multi-disciplinary rehabilitation in postacute COVID-19: an observational cohort study. *Eur J Phys Rehabil Med* **2021**, *57*, 189-198, doi:10.23736/s1973-9087.21.06549-7.
24. Soldati, A.B.; Almeida, C.; Lima, M.; Araujo, A.; Araujo-Leite, M.A.; Silva, M.T.T. Telephone Screening of Cognitive Status (TICS) in severe COVID-19 patients: Utility in the era of social isolation. *eNeurologicalSci* **2021**, *22*, 100322-100322, doi:10.1016/j.ensci.2021.100322.
25. Latronico, N.; Peli, E.; Calza, S.; Rodella, F.; Novelli, M.P.; Cella, A.; Marshall, J.; Needham, D.M.; Rasulo, F.A.; Piva, S. Physical, cognitive and mental health outcomes in 1-year survivors of COVID-19-associated ARDS. *Thorax* **2022**, *77*, 300-303, doi:10.1136/thoraxjnl-2021-218064.
26. Venturelli, S.; Benatti, S.V.; Casati, M.; Binda, F.; Zuglian, G.; Imeri, G.; Conti, C.; Biffi, A.M.; Spada, M.S.; Bondi, E.; et al. Surviving COVID-19 in Bergamo province: a post-acute outpatient re-evaluation. *Epidemiology and Infection* **2021**, *149*, e32, doi:10.1017/S0950268821000145.
27. Mazza, M.G.; Palladini, M.; De Lorenzo, R.; Magnaghi, C.; Poletti, S.; Furlan, R.; Ciceri, F.; Rovere-Querini, P.; Benedetti, F. Persistent psychopathology and neurocognitive impairment in COVID-19 survivors: Effect of inflammatory biomarkers at three-month follow-up. *Brain, Behavior, and Immunity* **2021**, *94*, 138-147, doi:https://doi.org/10.1016/j.bbi.2021.02.021.
28. Raman, B.; Cassar, M.P.; Tunnicliffe, E.M.; Filippini, N.; Griffanti, L.; Alfaro-Almagro, F.; Okell, T.; Sheerin, F.; Xie, C.; Mahmod, M.; et al. Medium-term effects of SARS-CoV-2 infection on multiple vital organs, exercise capacity, cognition, quality of life and mental health, post-hospital discharge. *EClinicalMedicine* **2021**, *31*, 100683, doi:https://doi.org/10.1016/j.eclinm.2020.100683.
29. Dressing, A.; Bormann, T.; Blazhenets, G.; Schroeter, N.; Walter, L.I.; Thurow, J.; August, D.; Hilger, H.; Stete, K.; Gerstacker, K.; et al. Neuropsychologic Profiles and Cerebral Glucose Metabolism in

Neurocognitive Long COVID Syndrome. *J Nucl Med* **2022**, *63*, 1058-1063, doi:10.2967/jnumed.121.262677.
30. van den Borst, B.; Peters, J.B.; Brink, M.; Schoon, Y.; Bleeker-Rovers, C.P.; Schers, H.; van Hees, H.W.H.; van Helvoort, H.; van den Boogaard, M.; van der Hoeven, H.; et al. Comprehensive Health Assessment 3 Months After Recovery From Acute Coronavirus Disease 2019 (COVID-19). *Clin Infect Dis* **2021**, *73*, e1089-e1098, doi:10.1093/cid/ciaa1750.
31. Monti, G.; Leggieri, C.; Fominskiy, E.; Scandroglio, A.M.; Colombo, S.; Tozzi, M.; Moizo, E.; Mucci, M.; Crivellari, M.; Pieri, M.; et al. Two-months quality of life of COVID-19 invasively ventilated survivors; an Italian single-center study. *Acta Anaesthesiologica Scandinavica* **2021**, *65*, 912-920, doi:https://doi.org/10.1111/aas.13812.
32. Del Brutto, O.H.; Wu, S.; Mera, R.M.; Costa, A.F.; Recalde, B.Y.; Issa, N.P. Cognitive decline among individuals with history of mild symptomatic SARS-CoV-2 infection: A longitudinal prospective study nested to a population cohort. *Eur J Neurol* **2021**, *28*, 3245-3253, doi:10.1111/ene.14775.
33. Group, T.W.C.f.t.C.S. Four-Month Clinical Status of a Cohort of Patients After Hospitalization for COVID-19. *JAMA* **2021**, *325*, 1525-1534, doi:10.1001/jama.2021.3331.
34. Rass, V.; Beer, R.; Schiefecker, A.J.; Kofler, M.; Lindner, A.; Mahlknecht, P.; Heim, B.; Limmert, V.; Sahanic, S.; Pizzini, A.; et al. Neurological outcome and quality of life 3 months after COVID-19: A prospective observational cohort study. *European Journal of Neurology* **2021**, *28*, 3348-3359, doi:https://doi.org/10.1111/ene.14803.
35. Almeria, M.; Cejudo, J.C.; Sotoca, J.; Deus, J.; Krupinski, J. Cognitive profile following COVID-19 infection: Clinical predictors leading to neuropsychological impairment. *Brain, Behavior, & Immunity - Health* **2020**, *9*, 100163, doi:https://doi.org/10.1016/j.bbih.2020.100163.
36. Cian, V.; De Laurenzis, A.; Siri, C.; Gusmeroli, A.; Canesi, M. Cognitive and Neuropsychiatric Features of COVID-19 Patients After Hospital Dismission: An Italian Sample. *Frontiers in Psychology* **2022**, *13*, doi:10.3389/fpsyg.2022.908363.
37. De Lorenzo, R.; Conte, C.; Lanzani, C.; Benedetti, F.; Roveri, L.; Mazza, M.G.; Brioni, E.; Giacalone, G.; Canti, V.; Sofia, V.; et al. Residual clinical damage after COVID-19: A retrospective and prospective observational cohort study. *PLOS ONE* **2020**, *15*, e0239570, doi:10.1371/journal.pone.0239570.
38. Walle-Hansen, M.M.; Ranhoff, A.H.; Mellingsæter, M.; Wang-Hansen, M.S.; Myrstad, M. Health-related quality of life, functional decline, and long-term mortality in older patients following hospitalisation due to COVID-19. *BMC Geriatrics* **2021**, *21*, 199, doi:10.1186/s12877-021-02140-x.
39. Patel, R.; Savrides, I.; Cahalan, C.; Doulatani, G.; O'Dell, M.W.; Toglia, J.; Jaywant, A. Cognitive impairment and functional change in COVID-19 patients undergoing inpatient rehabilitation. *Int J Rehabil Res* **2021**, *44*, 285-288, doi:10.1097/mrr.0000000000000483.
40. Manera, M.R.; Fiabane, E.; Pain, D.; Aiello, E.N.; Radici, A.; Ottonello, M.; Padovani, M.; Wilson, B.A.; Fish, J.; Pistarini, C. Clinical features and cognitive sequelae in COVID-19: a retrospective study on N=152 patients. *Neurological Sciences* **2022**, *43*, 45-50, doi:10.1007/s10072-021-05744-8.
41. Sardella, A.; Chiara, E.; Alibrandi, A.; Bellone, F.; Catalano, A.; Lenzo, V.; Quattropani, M.C.; Basile, G. Changesin Cognitive and Functional Status and in Quality of Life of Older Outpatients during the COVID-19 Pandemic. *Gerontology* **2022**, 1-6, doi:10.1159/000525041.
42. Abdelghani, M.; Atwa, S.A.; Said, A.; Zayed, N.E.; Abdelmoaty, A.A.; Hassan, M.S. Cognitive after-effects and associated correlates among post-illness COVID-19 survivors: a cross-sectional study, Egypt. *The Egyptian Journal of Neurology, Psychiatry and Neurosurgery* **2022**, *58*, 77, doi:10.1186/s41983-022-00505-6.
43. Aiello, E.N.; Radici, A.; Mora, G.; Pain, D. Cognitive phenotyping of post-infectious SARS-CoV-2 patients. *Neurological Sciences* **2022**, *43*, 4599-4604, doi:10.1007/s10072-022-06130-8.
44. Bolattürk, Ö.F.; Soylu, A.C. Evaluation of cognitive, mental, and sleep patterns of post-acute COVID-19 patients and their correlation with thorax CT. *Acta Neurologica Belgica* **2022**, doi:10.1007/s13760-022-02001-3.
45. Cecchetti, G.; Agosta, F.; Canu, E.; Basaia, S.; Barbieri, A.; Cardamone, R.; Bernasconi, M.P.; Castelnovo, V.; Cividini, C.; Cursi, M.; et al. Cognitive, EEG, and MRI features of COVID-19 survivors: a 10-month study. *Journal of Neurology* **2022**, *269*, 3400-3412, doi:10.1007/s00415-022-11047-5.

46. Guo, P.; Benito Ballesteros, A.; Yeung, S.P.; Liu, R.; Saha, A.; Curtis, L.; Kaser, M.; Haggard, M.P.; Cheke, L.G. COVCOG 2: Cognitive and Memory Deficits in Long COVID: A Second Publication From the COVID and Cognition Study. *Frontiers in Aging Neuroscience* **2022**, *14*, doi:10.3389/fnagi.2022.804937.
47. Henneghan, A.M.; Lewis, K.A.; Gill, E.; Kesler, S.R. Cognitive Impairment in Non-critical, Mild-to-Moderate COVID-19 Survivors. *Frontiers in Psychology* **2022**, *13*, doi:10.3389/fpsyg.2022.770459.
48. Serrano-Castro, P.J.; Garzón-Maldonado, F.J.; Casado-Naranjo, I.; Ollero-Ortiz, A.; Mínguez-Castellanos, A.; Iglesias-Espinosa, M.; Baena-Palomino, P.; Sánchez-Sanchez, V.; Sánchez-Pérez, R.M.; Rubi-Callejon, J.; et al. The cognitive and psychiatric subacute impairment in severe Covid-19. *Scientific Reports* **2022**, *12*, 3563, doi:10.1038/s41598-022-07559-9.
49. Becker, J.H.; Lin, J.J.; Doernberg, M.; Stone, K.; Navis, A.; Festa, J.R.; Wisnivesky, J.P. Assessment of Cognitive Function in Patients After COVID-19 Infection. *JAMA Network Open* **2021**, *4*, e2130645-e2130645, doi:10.1001/jamanetworkopen.2021.30645.
50. Ermis, U.; Rust, M.I.; Bungenberg, J.; Costa, A.; Dreher, M.; Balfanz, P.; Marx, G.; Wiesmann, M.; Reetz, K.; Tauber, S.C.; et al. Neurological symptoms in COVID-19: a cross-sectional monocentric study of hospitalized patients. *Neurological Research and Practice* **2021**, *3*, 17, doi:10.1186/s42466-021-00116-1.
51. Jaywant, A.; Vanderlind, W.M.; Alexopoulos, G.S.; Fridman, C.B.; Perlis, R.H.; Gunning, F.M. Frequency and profile of objective cognitive deficits in hospitalized patients recovering from COVID-19. *Neuropsychopharmacology* **2021**, *46*, 2235-2240, doi:10.1038/s41386-021-00978-8.
52. Albu, S.; Zozaya, N.R.; Murillo, N.; García-Molina, A.; Chacón, C.A.F.; Kumru, H. What's going on following acute covid-19? Clinical characteristics of patients in an out-patient rehabilitation program. *NeuroRehabilitation* **2021**, *48*, 469-480, doi:10.3233/nre-210025.
53. Ferrucci, R.; Dini, M.; Groppo, E.; Rosci, C.; Reitano, M.R.; Bai, F.; Poletti, B.; Brugnera, A.; Silani, V.; D'Arminio Monforte, A.; et al. Long-Lasting Cognitive Abnormalities after COVID-19. *Brain Sciences* **2021**, *11*, 235.
54. Johnsen, S.; Sattler, S.M.; Miskowiak, K.W.; Kunalan, K.; Victor, A.; Pedersen, L.; Andreassen, H.F.; Jørgensen, B.J.; Heebøll, H.; Andersen, M.B.; et al. Descriptive analysis of long COVID sequelae identified in a multidisciplinary clinic serving hospitalised and non-hospitalised patients. *ERJ Open Research* **2021**, *7*, 00205-02021, doi:10.1183/23120541.00205-2021.
55. Liu, Y.-H.; Wang, Y.-R.; Wang, Q.-H.; Chen, Y.; Chen, X.; Li, Y.; Cen, Y.; Xu, C.; Hu, T.; Liu, X.-D.; et al. Post-infection cognitive impairments in a cohort of elderly patients with COVID-19. *Molecular Neurodegeneration* **2021**, *16*, 48, doi:10.1186/s13024-021-00469-w.
56. Hellgren, L.; Birberg Thornberg, U.; Samuelsson, K.; Levi, R.; Divanoglou, A.; Blystad, I. Brain MRI and neuropsychological findings at long-term follow-up after COVID-19 hospitalisation: an observational cohort study. *BMJ Open* **2021**, *11*, e055164, doi:10.1136/bmjopen-2021-055164.
57. Méndez, R.; Balanzá-Martínez, V.; Luperdi, S.C.; Estrada, I.; Latorre, A.; González-Jiménez, P.; Feced, L.; Bouzas, L.; Yépez, K.; Ferrando, A.; et al. Short-term neuropsychiatric outcomes and quality of life in COVID-19 survivors. *J Intern Med* **2021**, *290*, 621-631, doi:10.1111/joim.13262.
58. Versace, V.; Sebastianelli, L.; Ferrazzoli, D.; Romanello, R.; Ortelli, P.; Saltuari, L.; D'Acunto, A.; Porrazzini, F.; Ajello, V.; Oliviero, A.; et al. Intracortical GABAergic dysfunction in patients with fatigue and dysexecutive syndrome after COVID-19. *Clinical Neurophysiology* **2021**, *132*, 1138-1143, doi:https://doi.org/10.1016/j.clinph.2021.03.001.
59. Woo, M.S.; Malsy, J.; Pöttgen, J.; Seddiq Zai, S.; Ufer, F.; Hadjilaou, A.; Schmiedel, S.; Addo, M.M.; Gerloff, C.; Heesen, C.; et al. Frequent neurocognitive deficits after recovery from mild COVID-19. *Brain Communications* **2020**, *2*, doi:10.1093/braincomms/fcaa205.
60. Zhou, H.; Lu, S.; Chen, J.; Wei, N.; Wang, D.; Lyu, H.; Shi, C.; Hu, S. The landscape of cognitive function in recovered COVID-19 patients. *Journal of Psychiatric Research* **2020**, *129*, 98-102, doi:https://doi.org/10.1016/j.jpsychires.2020.06.022.
61. Hadad, R.; Khoury, J.; Stanger, C.; Fisher, T.; Schneer, S.; Ben-Hayun, R.; Possin, K.; Valcour, V.; Aharon-Peretz, J.; Adir, Y. Cognitive dysfunction following COVID-19 infection. *J Neurovirol* **2022**, *28*, 430-437, doi:10.1007/s13365-022-01079-y.

62. Miskowiak, K.W.; Fugledalen, L.; Jespersen, A.E.; Sattler, S.M.; Podlekareva, D.; Rungby, J.; Porsberg, C.M.; Johnsen, S. Trajectory of cognitive impairments over 1 year after COVID-19 hospitalisation: Pattern, severity, and functional implications. *European Neuropsychopharmacology* **2022**, *59*, 82-92, doi:https://doi.org/10.1016/j.euroneuro.2022.04.004.
63. Amalakanti, S.; Arepalli, K.V.R.; Jillella, J.P. Cognitive assessment in asymptomatic COVID-19 subjects. *VirusDisease* **2021**, *32*, 146-149, doi:10.1007/s13337-021-00663-w.
64. Ortelli, P.; Ferrazzoli, D.; Sebastianelli, L.; Engl, M.; Romanello, R.; Nardone, R.; Bonini, I.; Koch, G.; Saltuari, L.; Quartarone, A.; et al. Neuropsychological and neurophysiological correlates of fatigue in post-acute patients with neurological manifestations of COVID-19: Insights into a challenging symptom. *Journal of the Neurological Sciences* **2021**, *420*, doi:10.1016/j.jns.2020.117271.
65. Tolentino, J.C.; Gjorup, A.L.T.; Schmidt, G.J.; Schmidt, S.L. Early attention impairment in a patient with COVID-19. *Psychiatry and Clinical Neurosciences* **2021**, *75*, 66-67, doi:https://doi.org/10.1111/pcn.13178.
66. Yesilkaya, U.H.; Sen, M.; Balcioglu, Y.H. COVID-19-related cognitive dysfunction may be associated with transient disruption in the DLPFC glutamatergic pathway. *Journal of Clinical Neuroscience* **2021**, *87*, 153-155, doi:https://doi.org/10.1016/j.jocn.2021.03.007.
67. Hellmuth, J.; Barnett, T.A.; Asken, B.M.; Kelly, J.D.; Torres, L.; Stephens, M.L.; Greenhouse, B.; Martin, J.N.; Chow, F.C.; Deeks, S.G.; et al. Persistent COVID-19-associated neurocognitive symptoms in non-hospitalized patients. *Journal of NeuroVirology* **2021**, *27*, 191-195, doi:10.1007/s13365-021-00954-4.
68. Whiteside, D.M.; Oleynick, V.; Holker, E.; Waldron, E.J.; Porter, J.; Kasprzak, M. Neurocognitive deficits in severe COVID-19 infection: Case series and proposed model. *The Clinical Neuropsychologist* **2021**, *35*, 799-818, doi:10.1080/13854046.2021.1874056.
69. Gautam, N.; Madathil, S.; Tahani, N.; Bolton, S.; Parekh, D.; Stockley, J.; Goyal, S.; Qureshi, H.; Yasmin, S.; Cooper, B.G.; et al. Medium-Term Outcomes in Severely to Critically Ill Patients With Severe Acute Respiratory Syndrome Coronavirus 2 Infection. *Clin Infect Dis* **2022**, *74*, 301-308, doi:10.1093/cid/ciab341.
70. Beaud, V.; Crottaz-Herbette, S.; Dunet, V.; Vaucher, J.; Bernard-Valnet, R.; Du Pasquier, R.; Bart, P.-A.; Clarke, S. Pattern of cognitive deficits in severe COVID-19. *Journal of Neurology, Neurosurgery & Psychiatry* **2021**, *92*, 567-568, doi:10.1136/jnnp-2020-325173.
71. Groiss, S.J.; Balloff, C.; Elben, S.; Brandenburger, T.; Müttel, T.; Kindgen-Milles, D.; Vollmer, C.; Feldt, T.; Kunstein, A.; Ole Jensen, B.-E.; et al. Prolonged Neuropsychological Deficits, Central Nervous System Involvement, and Brain Stem Affection After COVID-19—A Case Series. *Frontiers in Neurology* **2020**, *11*, doi:10.3389/fneur.2020.574004.
72. Negrini, F.; Ferrario, I.; Mazziotti, D.; Berchicci, M.; Bonazzi, M.; de Sire, A.; Negrini, S.; Zapparoli, L. Neuropsychological Features of Severe Hospitalized Coronavirus Disease 2019 Patients at Clinical Stability and Clues for Postacute Rehabilitation. *Archives of Physical Medicine and Rehabilitation* **2021**, *102*, 155-158, doi:https://doi.org/10.1016/j.apmr.2020.09.376.
